# Alteration of lung tissues proteins in birch pollen induced asthma mice before and after SCIT

**DOI:** 10.1371/journal.pone.0258051

**Published:** 2021-10-07

**Authors:** Zhijuan Xie, Haidan Sun, Xiaogang Li, Wei Sun, Jia Yin

**Affiliations:** 1 Department of Allergy, Peking Union Medical College Hospital, Chinese Academy of Medical Sciences and Peking Union Medical College, Beijing, P.R. China; 2 Beijing Key Laboratory of Precision Medicine for Diagnosis and Treatment on Allergic Diseases, Beijing, P.R. China; 3 Clinical Immunology Center, Chinese Academy of Medical Sciences, Beijing, P.R. China; 4 Core Facility of Instrument, Institute of Basic Medical Sciences, Chinese Academy of Medical Sciences, School of Basic Medicine, Peking Union Medical College, Beijing, P.R. China; 5 Department of Central laboratory, Peking Union Medical College Hospital, Peking Union Medical College and Chinese Academy of Medical Science, Beijing, P.R. China; University of Michigan Health System, UNITED STATES

## Abstract

Subcutaneous immunotherapy (SCIT) is a classic form of allergen-specific immunotherapy that is used to treat birch pollen induced allergic asthma. To investigate the underlying molecular mechanisms of SCIT, we aimed to profile lung samples to explore changes in the differential proteome before and after SCIT in mice with allergic asthma. Fresh lungs were collected from three groups of female BALB/c mice: 1) control mice, 2) birch pollen-induced allergic mice, and 3) birch pollen-induced allergic mice with SCIT. Tandem mass tag (TMT) labelling coupled with liquid chromatography-tandem mass spectrometry (LC-MS/MS) was used to analyze the lung proteome in the mice. Ingenuity pathway analysis (IPA) and Gene Ontology (GO) classification analysis were applied to identify differentially expressed proteins (DEPs) and crucial pathways. The screened DEPs were validated by immunohistochemistry analysis. A total of 317 proteins were upregulated and 184 proteins were downregulated in the asthma group compared to those of the control group. In contrast, 639 DEPs (163 upregulated and 456 downregulated proteins) were identified after SCIT in comparison with those of the asthma group. Among the 639 DEPs, 277 proteins returned to similar levels as those of the relative non-asthma condition. Bioinformatic analysis revealed that the 277 proteins played a significant role in the leukocyte extravasation signaling pathway. The leukocyte extravasation signaling pathway and related DEPs were of crucial importance in birch pollen SCIT.

## Introduction

Allergic asthma is a complex multifactorial disease that is mainly caused by the inhalation of various allergens, and affects 1–18% of the population in different countries [1]. The increasing morbidity of asthma over the years seriously affects patient quality of life and causes a heavy financial burden [2]. Birch pollen (*Betula platyphylla)* is a well-known cause of spring allergic asthma in northern China and Europe [3].

Precision medicine holds the view that “one size fits all” management is insufficient for individual allergic asthma patients [4, 5]. According to precision medicine, the European Academy of Allergy and Clinical Immunology (EAACI) recommended that subcutaneous immunotherapy (SCIT) was the only disease-modifying intervention to effectively control allergic symptoms, induce tolerant immune responses and reduce the usage of inhaled corticosteroids in allergic asthma patients [6, 7]. However, the mechanism of SCIT is not well understood. There is a pressing need to determine important SCIT specific pathways and biomarkers that provide evidence for precision medicine.

To better understand the mechanism of SCIT, multiple omics technologies have been used to identify clinical biomarkers associated with SCIT [8–10]. Proteins, functional components of cells that catalyze vital processes, are in the first line of the immune response to internal or external threat [11]. Pediatric asthma related proteomics studies have been carried out to gain insights into disease mechanisms and have laid a foundation for the identification of biomarkers, such as C-C motif ligand 5 [12, 13]. Previous proteomics studies mainly focused on the asthma exacerbation or a therapeutic response to systemic corticosteroids [9]. Many researchers have used 2D gel electrophoresis or protein microarray to profile serum proteins of asthma patients [14], which makes it difficult to reflect and explain the local pathological and immune changes of allergic asthma. Unfortunately, patient lung tissues were prohibited for research purposes based on ethical principles, and we therefore designed and developed animal models.

In this study, we use 10-plex TMT labelling coupled with LC-MS/MS to screen DEPs between two groups, comparing the asthma profiles to the control profiles and the asthma profiles to the SCIT profiles. The overlapping DEPs between asthma/control and asthma/SCIT are poorly studied but have great potential in helping researchers to understand and characterize these SCIT biomarkers.

## Materials and methods

### Animal models

Female BALB/c mice (5–6 weeks) were purchased from Beijing Vital River Laboratory Animal Technology Co in Beijing, China. This study was carried out in strict accordance with the recommendations in the Guide for the Care and Use of Laboratory Animals of the Peking Union Medical College Hospital Animal Ethics Committee (Beijing, China). The protocol was approved by the Committee on the Ethics of Animal Experiments of the Peking Union Medical College Hospital of Chinese Academy of Medical Sciences and Peking Union Medical College (Beijing, China) (Protocol Number: XHDW-2019-003). All surgery was performed under sodium pentobarbital anesthesia, and all efforts were made to minimize suffering. Previously, our group have reported on this mouse model and its SCIT protocols in detail [15]. There was no difference between birch pollen-induced allergic mice with PBS SCIT and birch pollen-induced allergic mice without SCIT.

**Fig 1:**
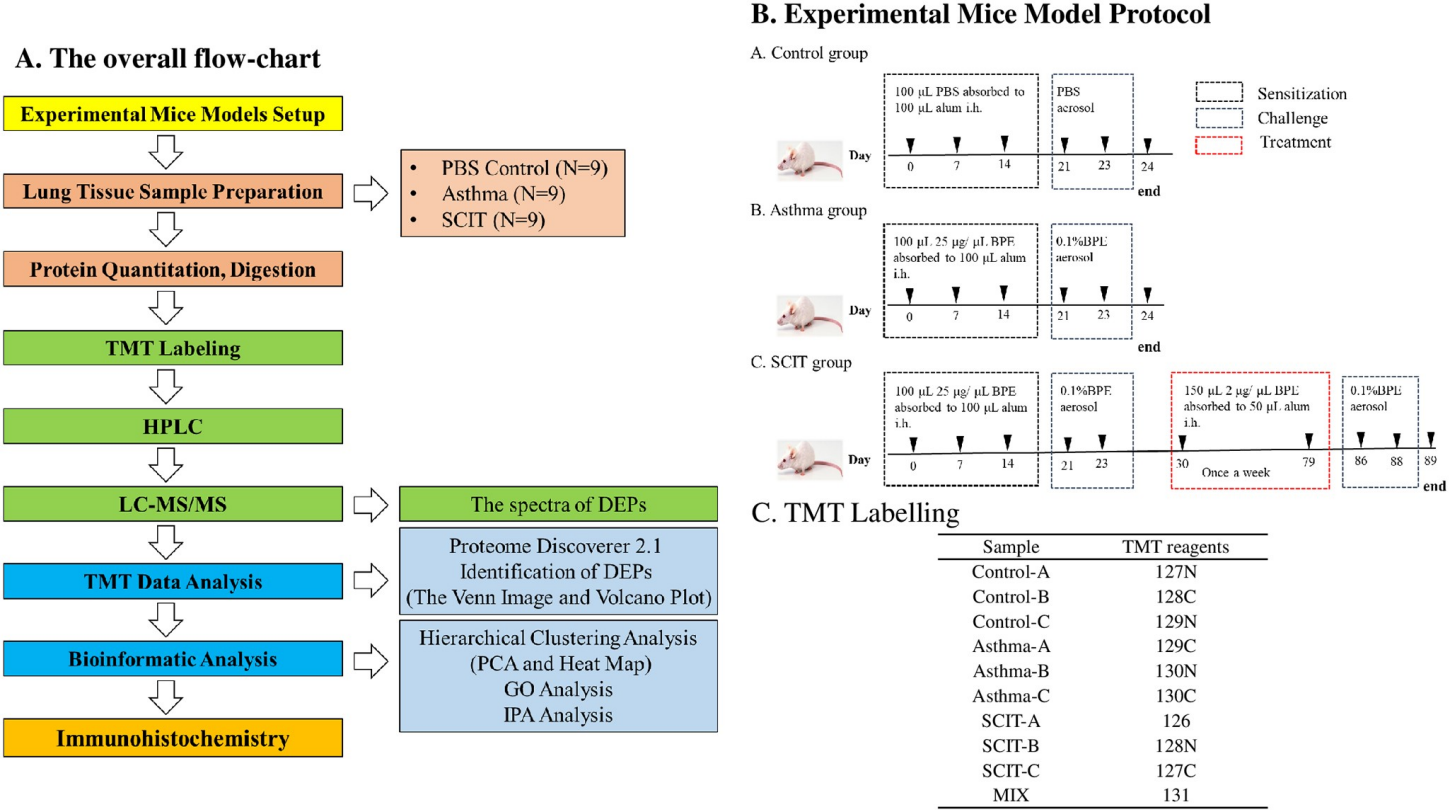
Study design. A. The overall flow-chart. B. Experimental mice model protocol. (1). The control group: The mice were primary sensitized with 100 μL (25 μg/μL) phosphate buffered saline (PBS) mixed with 100 μL alum (Imject Alum, Thermo, USA) by subcutaneous injection on days 0, 7 and 14. The mice received nebulized PBS in PBS 3 times for 30 min on days 21–23. On day 24, the mice were sacrificed for sample preparation. (2). The asthma group: The mice were primary sensitized with 100 μL (25 μg/μL) birch pollen extract (BPE) mixed with 100 μL alum (Imject Alum, Thermo, USA) by subcutaneous injection on days 0, 7 and 14. The mice received nebulized 0.1% BPE in PBS 3 times for 30 min on days 21–23. On day 24, the mice were sacrificed for sample preparation. (3). The SCIT group. C. TMT labelling: TMT-127N, TMT-128C, and TMT-129N were used for the PBS control group sample pools; TMT-129C, TMT-130N, and TMT-130C were used for the asthma group sample pools and TMT-126, TMT-128N, and TMT-127C were used for the SCIT group sample pools.

The overall workflow is shown in Fig 1A. The mice were primary sensitized with 100 μL (25 μg/μL) birch pollen extract (BPE) or phosphate buffered saline (PBS) mixed with 100 μL alum (Imject Alum, Thermo, USA) by subcutaneous injection on days 0, 7 and 14. The mice received nebulized PBS or 0.1% BPE in PBS 3 times for 30 min on days 21–23. On day 24, the mice were anesthetized by intraperitoneal injection of 0.3–0.6 ml 1% pentobarbital until the pain reflex disappeared, then sacrificed for sample preparation (Fig 1B). The SCIT group mice were sensitized and challenged in the same manner as the asthma group and then subcutaneously injected with 150 μL (2 μg/μL)) BPE adsorbed to 50 μL alum on days 30, 37, 44, 51, 58, 65, 72 and 79. On days 86–88, the mice received nebulized 0.1% BPE in PBS for 30 min per day before they were killed, and the samples were collected (Fig 1B). We gave the mice euthanasia by anesthesia overdose. The evidence of euthanasia based on moribund appearance. The pain for the mice and method to calm is mild, transient or without pain. Details were shown in S1 File.

### Inflammatory cells in bronchoalveolar lavage fluids (BALF)

Inflammation cells in BALF were collected by a tracheal cannula and then analyzed by the Siemens automatic blood analyzer (ADVIA2120). Details were shown in S1 File.

### Lung tissue sample preparation

After the collection of BALF, the lung tissue samples were collected for proteomics analysis, histological examination and Immunohistochemistry (IHC) test.

### Histological examination

To evaluate the infiltration of inflammatory cells around the airways and blood vessels, the lung tissues were fixed with 10% formaldehyde and embedded in paraffin and then cut into slices for staining with hematoxylin & eosin (H&E).

### Immunohistochemistry (IHC)

IHC was performed on formalin-fixed and paraffin-embedded lung tissues strictly according to the protocols. The results were evaluated by the H-score of the whole stained sections. The H-score is a semi-quantitative histological scoring method that transfers the number of positive cells and the staining intensity of each section into corresponding values, as previously described [16]. Details were shown in S1 File.

### Protein digestion and TMT labeling

The experimental procedure for Thermo Scientific^™^ Tandem Mass Tag^™^ (TMT^™^) reagent labeling was performed strictly according to the manufacturer’s instructions. TMT-127N, TMT-128C, and TMT-129N were used for the PBS control group sample pools; TMT-129C, TMT-130N, and TMT-130C were used for the asthma group sample pools and TMT-126, TMT-128N, and TMT-127C were used for the SCIT group sample pools. A mixed sample pool for normalization was prepared from each individual sample pool and labeled with TMT-131 (Details shown in Fig 1C and S1 File).

### High performance liquid chromatography (HPLC)

Details were shown in S1 File.

### LC-MS/MS

Details were shown in S1 File.

### TMT data analysis

Details were shown in S1 File.

### Bioinformatics analysis

Details were shown in S1 File.

### Statistical analyses

All experimental data are presented as the mean ± SD. Statistical differences were assessed with the Mann–Whitney U test or Student’s t-test. The analyses were performed by using the statistical software SPSS 19.0 and GraphPad Prism 5.0. All tests were two-tailed and *P*-values < 0.05 were considered statistically significant.

## Results

### Study design

The overall workflow is shown in Fig 1A. We designed an experimental approach to examine changes in the proteome before and after SCIT. Moreover, we sought biomarkers among the DEPs that overlapped between asthma/control and asthma/SCIT. The control group (N = 9), asthma group (N = 9) and SCIT group (N = 9) samples were collected for protein quantitation and digestion, and then 10-plex TMT labeling reagents were randomly used to label the mixed digested peptide sample pools. The peptides mixture was analyzed by LC-MS/MS and raw data were explained with bioinformatics software. The proteomics results were further verified by immunohistochemistry analysis.

### Assessment of birch pollen induced asthma and SCIT efficacy in mice

**Fig 2:**
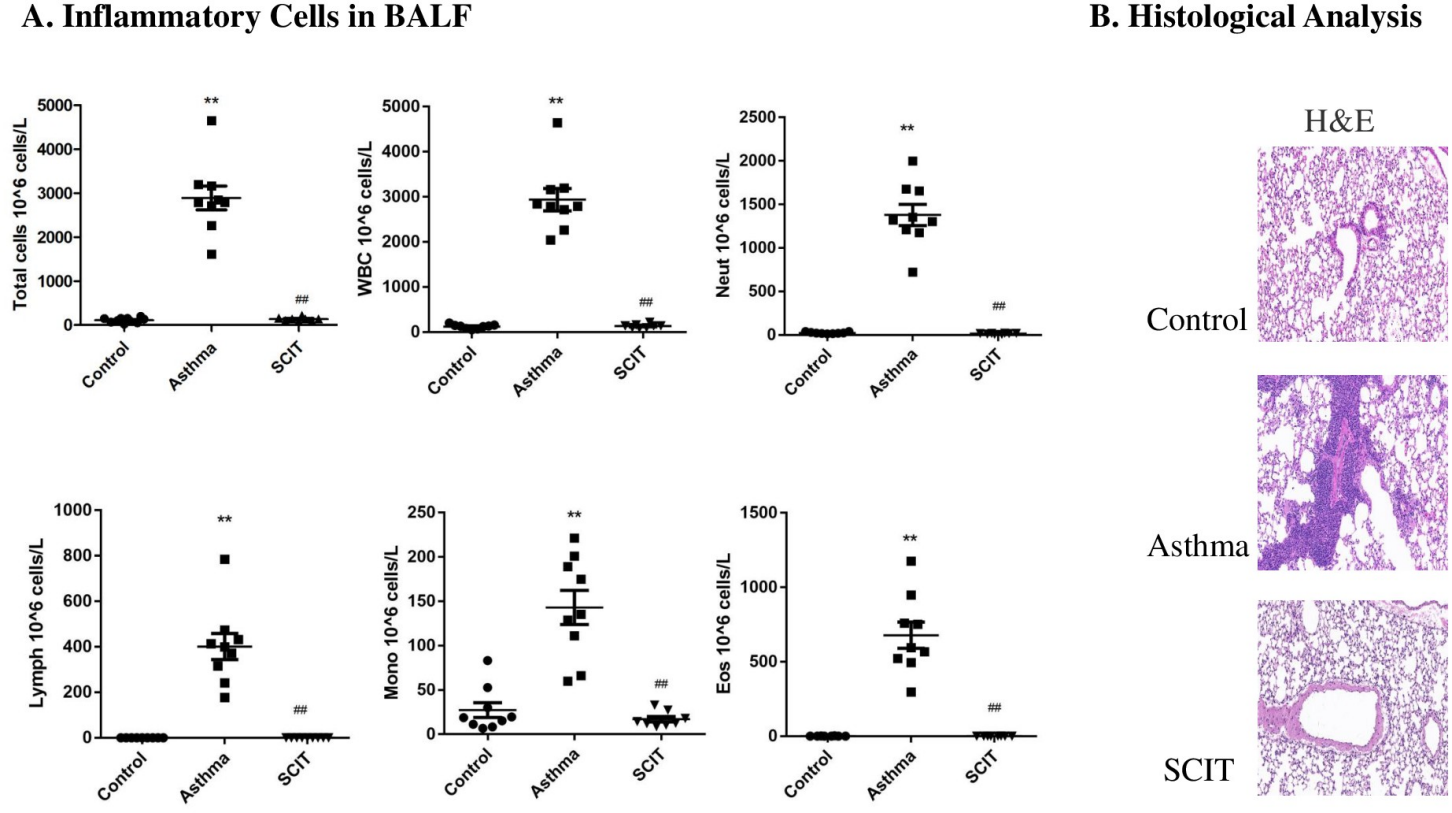
SCIT therapy reduces inflammatory responses in the lungs of birch pollen induced allergic mice. A. Inflammatory cells in BALF. Total cells; WBC, white blood cell; Neut, neutrophils; Lymph, lymphocytes; Mono, monocytes; Eos, eosinophils. ** represented the asthma group vs. the control group, *P* < 0.01; ^##^ represented the asthma group vs. the SCIT group, *P* < 0.01. B. Histological Analysis: Lung specimens were stained with H&E staining. Inflammation scores were shown as above.

The number of inflammatory cells in the BALF (total cells, neutrophils, lymphocytes, monocytes and eosinophils) all increased in the asthma group compared with that of the control group and then decreased significantly after SCIT (Fig 2A). Histological analyses showed that asthmatic lungs had more peribronchial infiltration of inflammatory cells than other groups (Fig 2B).

### Hierarchical clustering analysis

**Fig 3:**
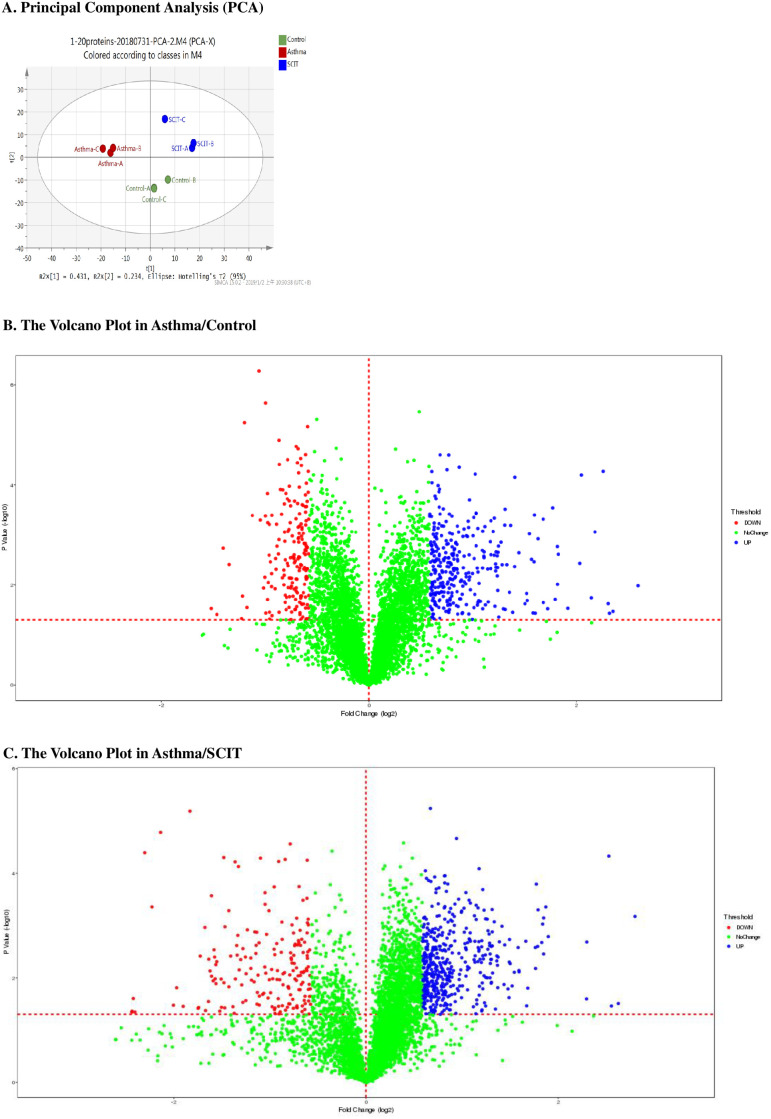
Hierarchical clustering analysis reveals differentially expressed protein profiles proteins between groups. A. Principal component analysis (PCA) showed obvious segregation and aggregation among the three groups, suggesting that our samples were of good quality for further proteomic analysis. B. The volcano plot indicated that the up-regulate (shown in blue) and down-regulate expression (shown in red) proteins of asthma group in comparation with the normal control group (shown in green). C. The volcano plot indicated that the up-regulate (shown in blue) and down-regulate expression (shown in red) proteins of asthma group in comparation with the SCIT group (shown in green).

In total, we identified 5923 proteins containing at least two unique peptides. Principal component analysis (PCA) of the 5923 proteins showed obvious segregation and aggregation among the three groups (Fig 3A). All the groups had specific characteristics and consistency, suggesting that our samples were of good quality for further proteomic analysis. The volcano plot showed the upregulate (shown in blue) and downregulate (shown in red) proteins in the asthma group in comparison with those of the control group (shown in green) (Fig 3B). Moreover, the volcano plot of asthma/SCIT is shown in Fig 3C.

### Screening differentially expressed proteins (DEPs)

**Fig 4:**
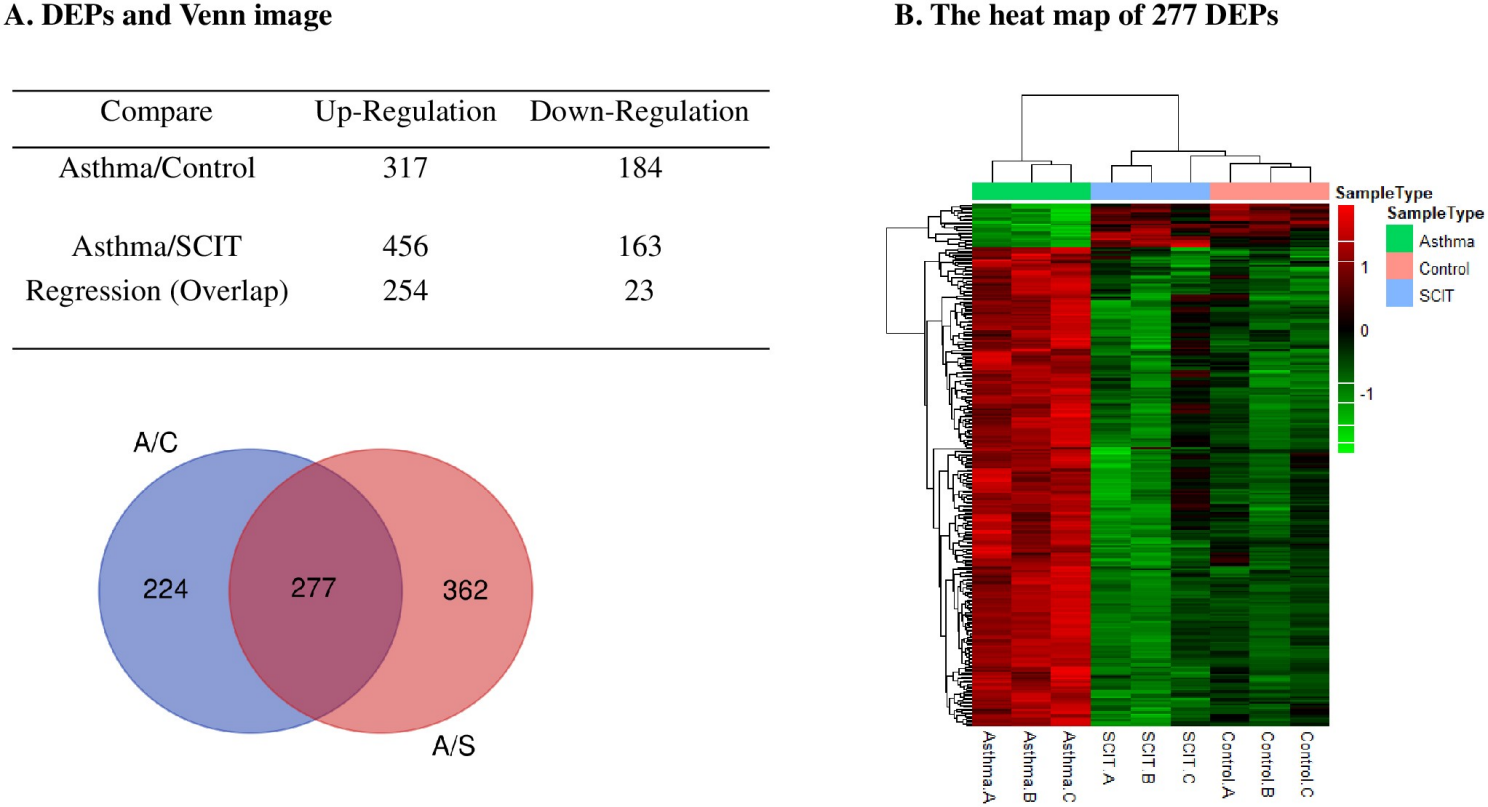
Identification of differentially expressed proteins before and after SCIT. A. Identification of differentially expressed proteins (DPEs), the Venn image; There were 501 differentially expressed proteins between the control group and the asthma group, of which 317 proteins were upregulated and 184 were proteins downregulated in the asthma group (shown in blue). 639 proteins (456 downregulated and 163 upregulated proteins in the asthma group) were differentially expressed after SCIT (shown in red). B. The heat map of 277 regression DEPs. The 277 overlapping proteins were significantly changed in the asthma condition compared with those of the control condition and then returned to levels similar to the related nondisease condition (just as the control condition) after receiving SCIT.

There were 501 differentially expressed proteins between the control group and the asthma group, of which 317 proteins were upregulated and 184 were proteins downregulated in the asthma group. Additionally, 639 proteins (456 downregulated and 163 upregulated proteins in the asthma group) were differentially expressed after SCIT (Fig 4A). Interestingly, 277 proteins (254 were upregulated and 23 were downregulated in the asthma group) overlapped between the asthma/control condition and asthma/SCIT condition (Fig 4A). The 277 overlapping proteins were significantly changed in the asthma condition compared with those of the control condition and then returned to levels similar to the related nondisease condition (just as the control condition) after receiving SCIT. The heat map of 277 DEPs were shown in Fig 4B. We hypothesize that the key biomarker or drug targets of SCIT may exist among these proteins. Furthermore, detailed information on the 277 DEPs is shown in S1 Table. Of the 277 regression proteins, at least 39 proteins (e.g, C-C motif chemokine 6) have been previously reported to be strongly associated with lung diseases (S2 Table), which provides strong support for the reliability of our MS data.

### Gene ontology classification analysis (GO analysis)

To analyze the 277 regression DEPs, biological process was mainly related to the cellular process (GO:0009987) while molecular function was related to catalytic activity (GO:0003824). cellular component pointed to the cell part (GO: 0044464) (S1A–S1C Fig) according to GO analysis. The whole mouse protein classes are shown as references to their related categories (S2A–S2D Fig).

### Ingenuity pathway analysis (IPA)

**Fig 5:**
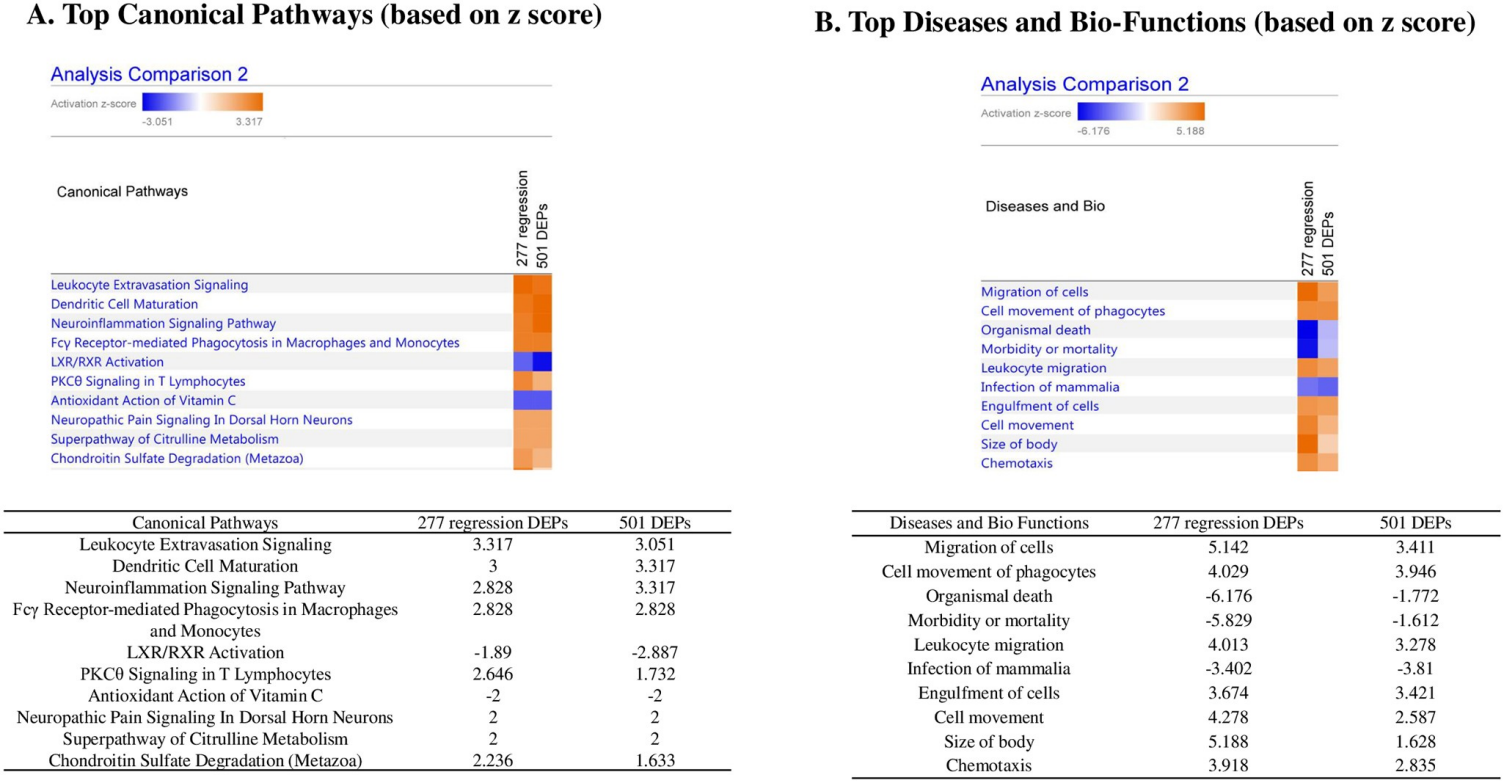
The Ingenuity pathway analysis (IPA) showed crucial pathway related with SCIT. A. Top 10 canonical pathways of 501 DEPs in asthma/control and 277 regression DEPs were listed in the table based on z-scores. B. Top 10 diseases and biofunctions of 501 DEPs in asthma/control and 277 regression DEPs were listed in the table based on z-scores.

IPA was used to list the top 10 activated canonical pathways in 277 regression DEPs and 501 DEPs based on z-scores (Fig 5A). The leukocyte extravasation signaling pathway showed the most significantly positive response, with a z-score of 3.317 in 277 regression DEPs and 3.051 in 501 DEPs. This suggests that leukocyte inflammatory responses are crucial in the mechanism of allergic asthma and SCIT. Of the diseases and biofunctions in IPA, the top biofunctions were Migration of cells (z-score 5.142), Cell movement of phagocytes (z-score 4.029), Leukocyte migration (z-score 4.013), Cell movement (z-score 4.278) and Chemotaxis (z-score 3.918) (Fig 5B). These results were strongly associated with the migration of inflammatory cells, especially leukocytes. Taken together, we noticed that leukocyte extravasation signaling pathway was closely connected with BPE-induced asthma and SCIT.

**Fig 6:**
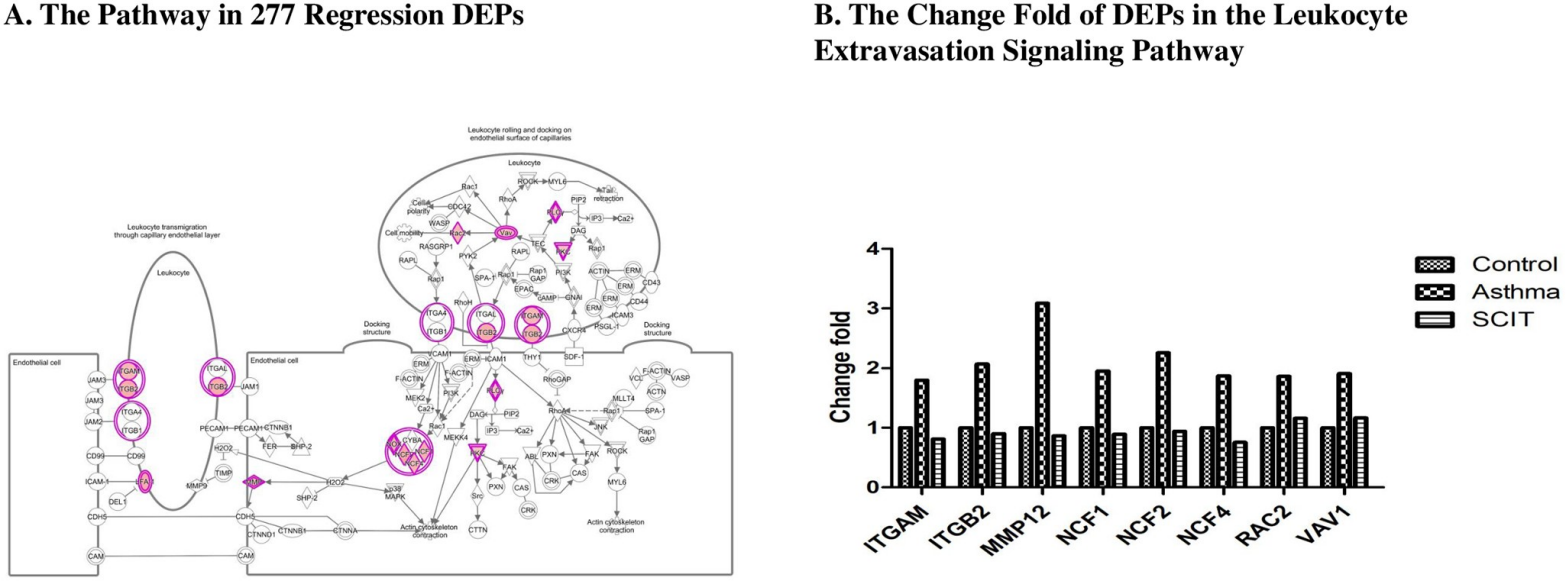
The leukocyte extravasation signaling assigned by the IPA software map. Each line suggested protein-protein interaction. Solid line indicated direct interaction while dashed line means indirect interaction. Red colors pointed to the up-regulation of the expression of DEPs in asthma condition. A. The leukocyte extravasation signaling pathway of 277 regression DEPs. B. The change fold of DEPs in the leukocyte extravasation signaling pathway based on the protein’s abundances.

Then, we constructed a complete leukocyte extravasation signaling pathway image with the help of IPA software (Fig 6A). The levels of most proteins (ITGAM, ITGB2, MMP12, NCF1, NCF2, NCF4, RAC2 and Vav1) were higher in the asthma condition (shown in red) than those in the control condition or after SCIT condition. The change fold clearly demonstrated that the protein abundances of those DEPs increased in asthma condition and then decreased after SCIT (Fig 6B). The spectra of these DEPs are shown in S3 Fig. All the information on the DEPs in the leukocyte extravasation signaling pathway were listed in S3 Table.

### Immunohistochemistry (IHC) results

**Fig 7:**
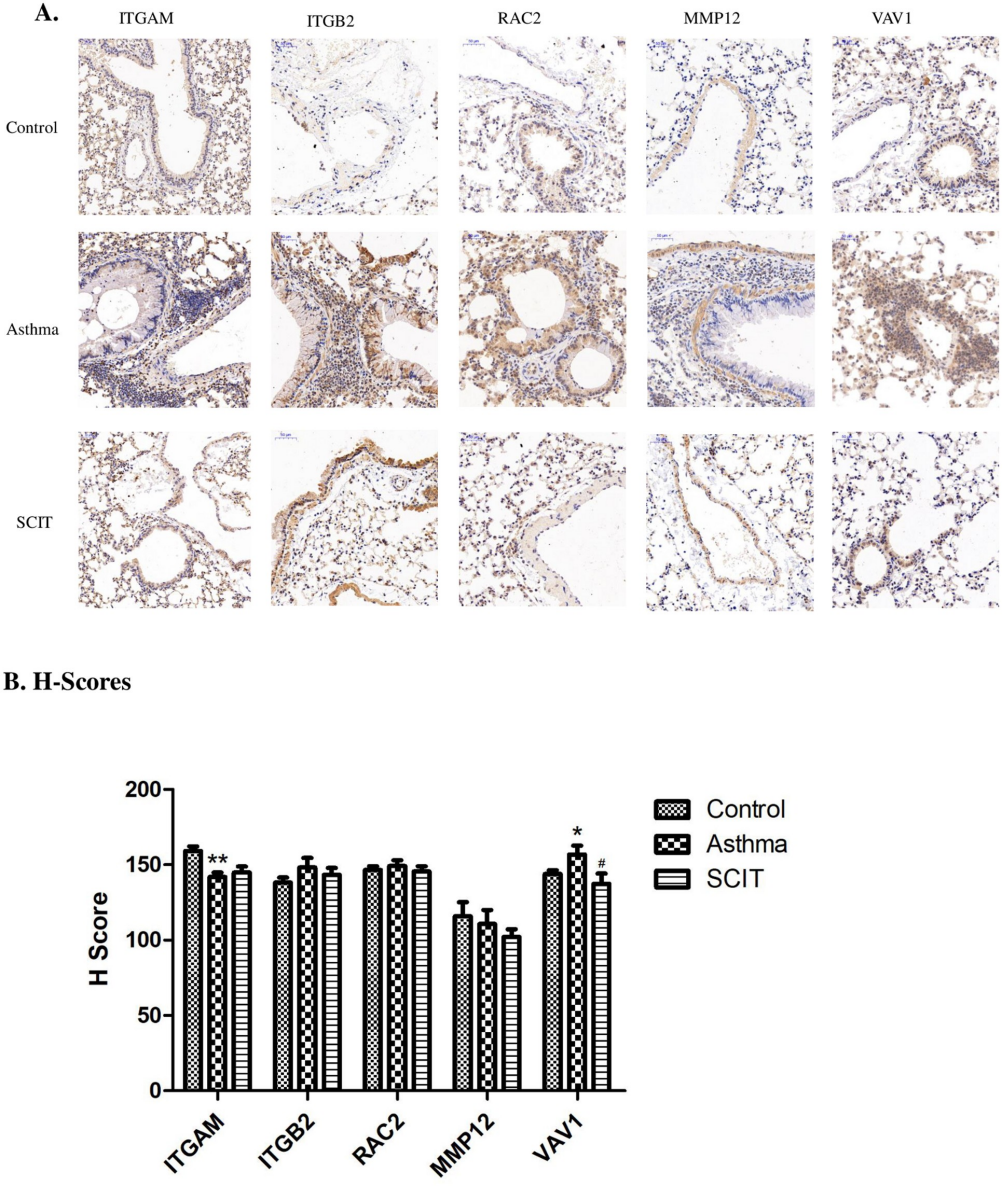
IHC results. A. IHC exhibited the expression of proteins in the leukocyte extravasation signaling pathway on formalin-fixed and paraffin-embedded mice lung tissues. Original magnification, × 200. B. H-scores representing IHC results were shown in the section. The histogram was shown as means ± SD, ** represented the asthma group vs. the control group, *P* < 0.01; * represented the asthma group vs. the control group, *P* < 0.05; ^#^ represented the asthma group vs. the SCIT group, *P* < 0.05.

The proteins (ITGAM, ITGB2, MMP12, RAC2 and Vav1) identified by proteomics in the leukocyte extravasation signaling pathway were chosen for verification. IHC was performed to detect the expression levels of these proteins in lung tissues of the three different groups. The results were calculated and analyzed based on the H-score of the whole lung tissues. As shown in Fig 7A, the expression levels (the staining intensity) of ITGB2, RAC2 and Vav1 were higher in the asthma group than other groups. The H-scores of Vav1 increased significantly in asthma condition and then decreased significantly after SCIT, supporting the above MS findings (Fig 7B).

## Discussion

SCIT has been used for more than 100 years as the disease-modifying therapy for allergic asthma. To compare changes in the proteome of lung tissues before and after SCIT, we designed mouse experiments. First, the control and the asthma samples were compared to obtain DEPs before SCIT. Then, we compared the asthma samples with the SCIT samples to obtain the DEPs after SCIT. Bioinformatic analysis pinpointed the 277 overlapping proteins were highly associated with leukocyte extravasation signaling pathway.

Leukocyte extravasation is the process by which leukocytes migrate from peripheral blood into local lung tissues in response to pollen allergens with the help of multiple adhesion molecules on the endothelium and their ligands on leukocytes [17]. The process requires multiple chemokines. Our research demonstrated that leukocyte extravasation was strongly influenced by SCIT. First, we found leukocytes (neutrophils, lymphocytes, basophils and eosinophils) recruited in BALF of allergic mice and these cells decreased significantly after SCIT. Recruited eosinophils from the circulation become activated and extravasated into the extracellular matrix of the airways, releasing active proteins to exacerbate airway inflammation and lung damage, which eventually lead to asthma [18, 19]. Then, leukocyte related chemokines such as CXC-chemokine ligand 8 (CXCL8), CXCL10, CC-chemokine ligand 2 (CCL2), CCL4 and CCL5 were detected in the 277 regression DEPs [20, 21]. SCIT decreased the level of chemokines and then partly inhibited leukocytes migrate from micro-vessels to local inflammatory microenvironment. Thirdly, according to IPA, the bio-function of the 277 regression DEPs in leukocyte extravasation signaling were highly related to cell migration.

To our surprise, many DEPs (ITGAM, ITGB2, MMP12, NCF1, NCF2, NCF4, RAC2 and Vav1) in the leukocyte extravasation signaling pathway increased in asthma condition then significantly decreased after SCIT. ITGAM, also named leukocyte adhesion receptor MO1 or CD11b, is a transmembrane glycoprotein, and ITGB2 (Integrin Beta-2) is usually recognized as CD18. Integrin ITGAM/ITGB2 is a receptor for fibrinogen, factor X and ICAM1 in leukocytes to regulate adhesion and transmigration [22, 23]. Integrin ITGAM/ITGB2 (αMβ2), a principal cell-surface proteins, is essential for the activation of blood eosinophils, recruitment to the airway, and finally the contribution to airway remodeling [24]. Integrin ITGAM/ITGB2 mediates eosinophils adhesion and extravasation though vascular cell adhesion molecule-1 (VCAM-1), intercellular adhesion molecule-1 (ICAM-1) and vitronectin [25]. Integrin conformational states are potentially correlated with asthma severity. It was reported that the ITGAM expression level on eosinophils was higher in the allergy season, but had no differences between asthma patients and healthy controls [26]. Integrin mediates the leukocyte extravasation depending on its activation state rather than its expression level [24].

MMP-12 is macrophage metalloelastase or matrix metalloproteinase-12, existing in macrophages and bronchial epithelial cells. MMP-12 is involved in influencing airway remodeling by mediating pathological degradation of the extracellular matrix and controlling the accumulation of eosinophils in response to allergen exposure [27]. The expression of MMP-12 was required for airway eosinophilia in OVA-induced allergic murine model [28]. In MMP-12 KO mice, the numbers of eosinophils were reduced in BALF compared with those of healthy controls, suggesting that MMP12 is key in the development of eosinophil extravasation. The risk *MMP12* gene variant has been reported to be highly related to the severity of asthma in patients and MMP-12 inhibitors may be considered as a potential therapeutic targets of asthma [29]. In fatal asthma, the expression of MMP-12 increased in airway smooth muscle in large airways compared with that of controls [30].

The Rho subfamily of guanine triphosphatases (GTPases) plays a role in regulating actin cytoskeletal rearrangement, and is composed of RAC1, RAC2 and RAC3. RAC2 is a typical small molecular G signaling protein expressed in hematopoietic cells [31]. RAC2 regulates cellular functions by switching from the inactive guanosine diphosphate (GDP)-bound state to the active guanosine triphosphate (GTP)-bound state [32]. In the pathogenesis of asthma, blood neutrophils migrate to the inflammation microenvironment of lung tissues in response to chemotactic signaling. Neutrophils induce a series of responses from the respiratory burst to generate reactive oxygen species (ROS), which eventually cause tissue damage [33]. RAC2 controls excessive reactive oxygen species (including superoxide, singlet O_2_, H_2_O_2_) generation by regulating nicotinamide adenine dinucleotide phosphate (NADPH) oxidase [34]. NADPH oxidase is responsible for respiratory bursts. Moreover, NCF1 (neutrophil cytosol factor 1), NCF2 (neutrophil cytosol factor 2) and NCF4 (neutrophil cytosol factor 4), which were identified in our proteomics study, are also associated with the activation of latent NADPH oxidase [35]. Moreover, RAC2 is important for eosinophils to migrate and release cytotoxic mediators during asthma [36]. In our study, we observed that RAC2 expression was closely connected with SCIT as a potential biomarker.

Vav1, known as proto-oncogene vav, is a phosphoprotein that is widely expressed in hematopoietic cells in lung tissues and affects the Rho/Rac family of GTPases in cell proliferation. Vav1 is an important regulator of T cell activation related cytoskeletal rearrangements [37]. Vav1 is involved in cell signaling and protein-protein interactions [38]. The IHC results showed that the expression of Vav1 in asthmatic mouse lung tissues was significantly higher than that in the other mouse groups, which was consistent with the proteomics findings. To the best of our knowledge, it is the first study to indicate that Vav1 may serve as a novel biomarker of SCIT. Combined with these observations, we unveiled parts of the underlying mechanism of SCIT and introduced some potential biomarkers for treating asthma disorders.

In conclusion, we used Chinese birch pollen induced allergic mice to investigate potential biomarkers of SCIT by profiling the proteomes. The leukocyte extravasation signaling pathway and related proteins (especially VAV1) may contribute to the SCIT mechanism and benefit the precision medicine.

## Supporting information

S1 Fig GO analysis of 277 regression DEPs. A. The molecular function (MF). B. The biological process (BP). C. The cellular component (CC).
(TIF)

S2 Fig GO analysis: The whole mouse protein class as reference. (TIF)

S3 Fig The spectra of these DEPs in the leukocyte extravasation signaling pathway. MS/MS data of represented peptides proteins (ITGAM, ITGB2, MMP12, NCF1, NCF2, NCF4, RAC2 and Vav1) were shown. A. The spectra of ITGB2 and MMP12. B. The spectra of VAV1 and NCF4. C. The spectra of NCF1 and RAC2. D. The spectra of NCF2 and ITGAM.
(TIF)

S1 Table Significantly up- and down- regulated 277 DEPs with *P* value ≤0.05 and fold change≥1.5 or ≤0.67 both in asthma/control and asthma/SCIT. (PDF)

S2 Table The 277 regression DEPs and previous reports associated with lung diseases. (PDF)

S3 Table The details of the DEPs in the leukocyte extravasation signaling. (PDF)

S1 File (DOCX)

S1 Checklist (PDF)

## Abbreviations

BALF: Bronchoalveolar Lavage Fluid
BP: Biological Process
BPE: Birch Pollen Extract
BSA: Bovine Serum Albumin
CC: Cellular Component
DEPs: Differentially Expressed Proteins
EAACI: European Academy of Allergy and Clinical Immunology
FDR: False Discovery Rate
GO: Gene Ontology classification analysis
HCA: Hierarchical Clustering Analysis
H-score: Histochemistry score
IHC: Immunohistochemistry
IPA: Ingenuity Pathway Analysis
MF: Molecular Function
PBS: Phosphate Buffered Saline
PCA: Principal Component Analysis
SCIT: Subcutaneous Immunotherapy
