# Supplementary figures and images for: Alteration of lung tissues proteins in birch pollen induced asthma mice before and after SCIT
Source: PLoS One. 2021 Oct 7;16(10):e0258051. doi: 10.1371/journal.pone.0258051 (PMC8496856; doi:10.1371/journal.pone.0258051)

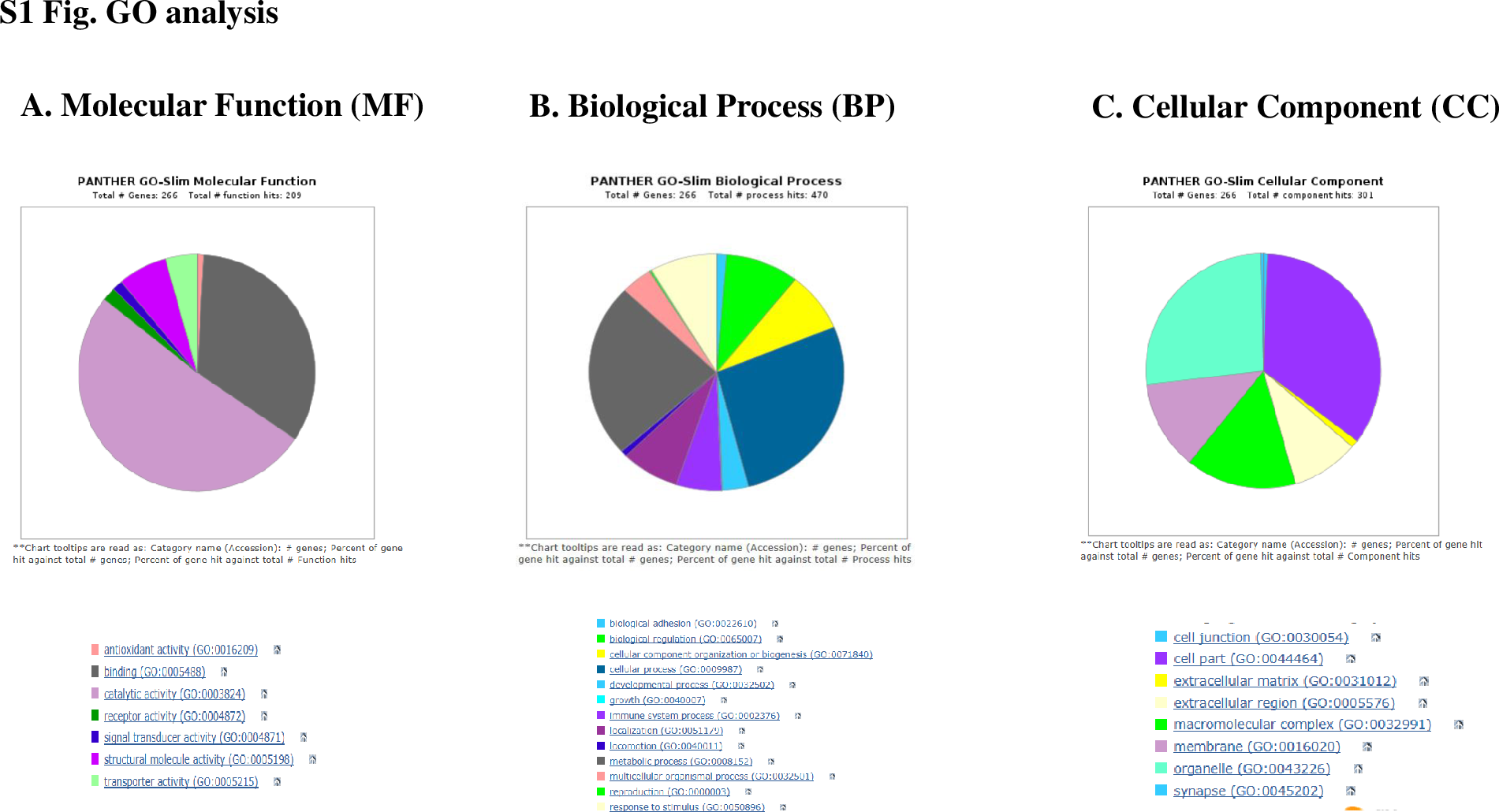

Supplement: S1 Fig — A. The molecular function (MF). B. The biological process (BP). C. The cellular component (CC). (TIF) [file pone.0258051.s001.tif]

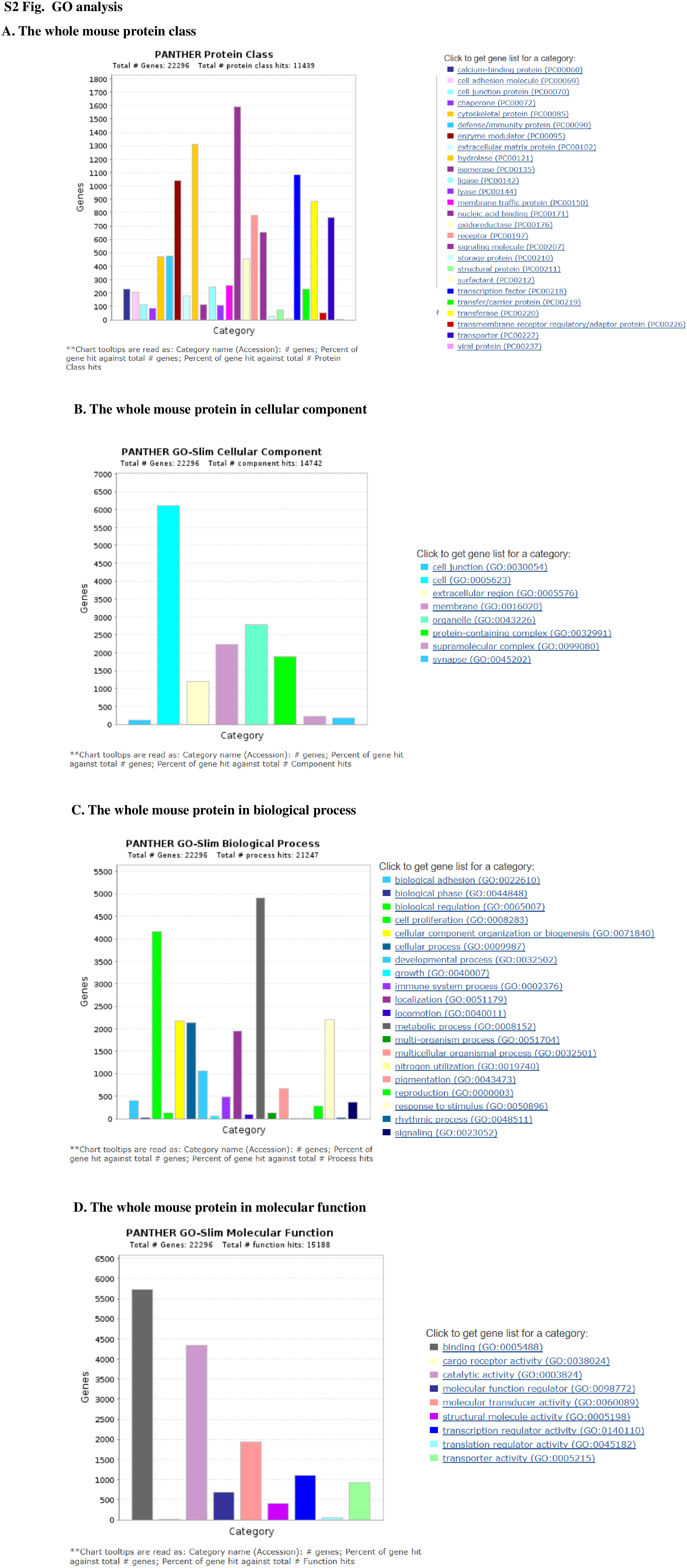

Supplement: S2 Fig — (TIF) [file pone.0258051.s002.tif]

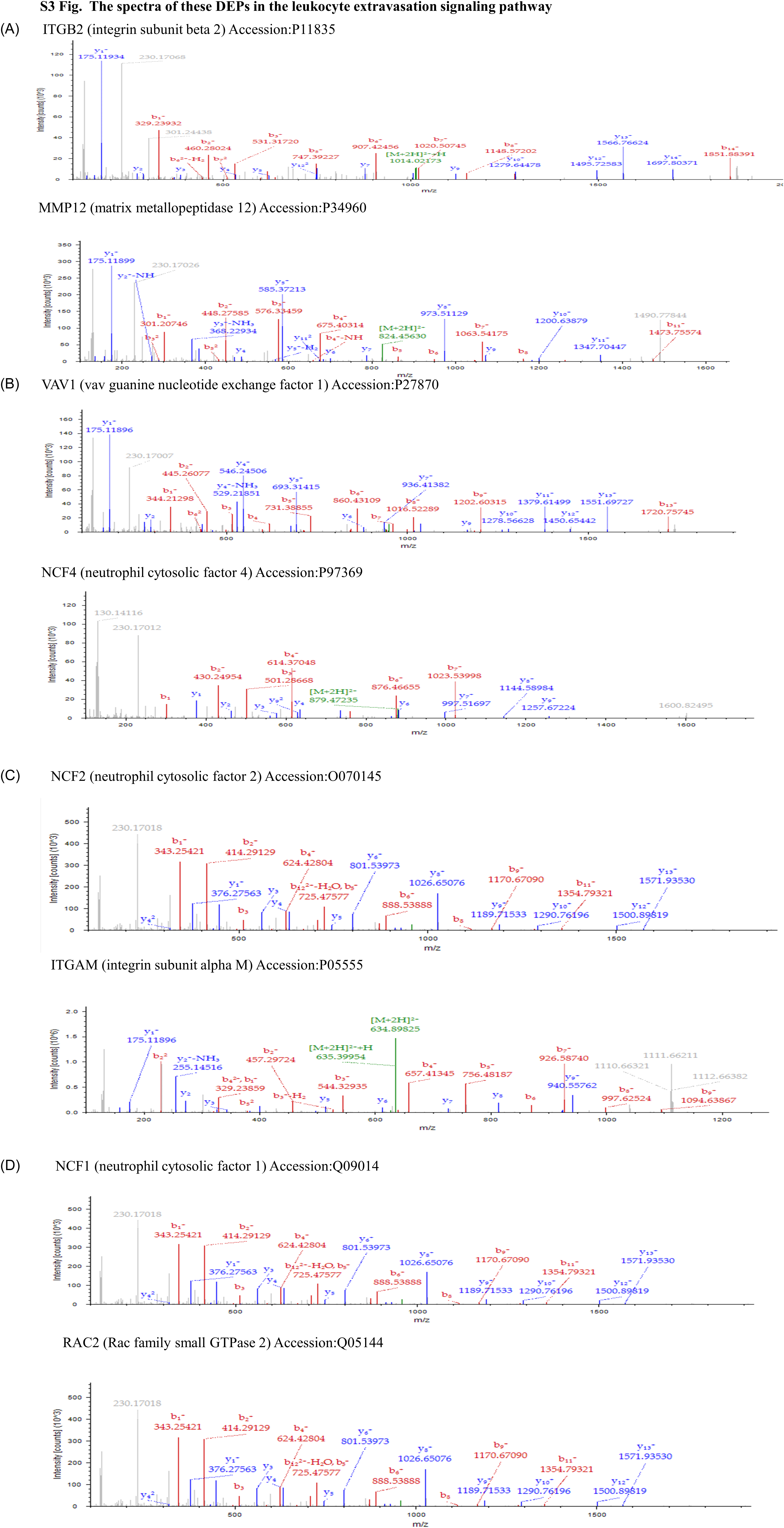

Supplement: S3 Fig — MS/MS data of represented peptides proteins (ITGAM, ITGB2, MMP12, NCF1, NCF2, NCF4, RAC2 and Vav1) were shown. A. The spectra of ITGB2 and MMP12. B. The spectra of VAV1 and NCF4. C. The spectra of NCF1 and RAC2. D. The spectra of NCF2 and ITGAM. (TIF) [file pone.0258051.s003.tif]
