# Supplementary material for: Alteration of lung tissues proteins in birch pollen induced asthma mice before and after SCIT
Source: PLoS One. 2021 Oct 7;16(10):e0258051. doi: 10.1371/journal.pone.0258051 (PMC8496856; doi:10.1371/journal.pone.0258051)
Supplement: S1 Table — (PDF) [file pone.0258051.s004.pdf]

S1 Table. Significantly up- and down- regulated 277 DEPs with *P* value ≤0.05 and fold change≥1.5 or ≤0.67 both in asthma/control and asthma/SCIT

Unique Accession	Description	Unique peptides	P-value A/C	Fold change A/C	P-value A/S	Fold change A/S
A6H584	Collagen alpha-5(VI) chain OS=Mus musculus GN=Col6a5 PE=1 SV=4	58	0.02	3.21	0.005	2.83
Q9WTI7-3	Isoform 3 of Unconventional myosin-Ic OS=Mus musculus GN=Myo1c	53	0.00	0.38	0.008	0.42
Q99PL5	Ribosome-binding protein 1 OS=Mus musculus GN=Rrbp1 PE=1 SV=2	46	0.01	2.00	0.009	1.96
P20152	Vimentin OS=Mus musculus GN=Vim PE=1 SV=3	46	0.01	1.60	0.013	1.62
P27773	Protein disulfide-isomerase A3 OS=Mus musculus GN=Pdia3 PE=1 SV=2	44	0.00	1.70	0.003	1.86
P20029	78 kDa glucose-regulated protein OS=Mus musculus GN=Hspa5 PE=1 SV=3	43	0.00	2.17	0.000	2.48
P08071	Lactotransferrin OS=Mus musculus GN=Ltf PE=1 SV=4	41	0.02	1.54	0.013	1.74
Q9D7Z6	Calcium-activated chloride channel regulator 1 OS=Mus musculus GN=Clca1 PE=1 SV=2	41	0.00	4.14	0.000	3.66
P08113	Endoplasmin OS=Mus musculus GN=Hsp90b1 PE=1 SV=2	40	0.00	1.55	0.001	1.87
Q61233	Plastin-2 OS=Mus musculus GN=Lcp1 PE=1 SV=4	40	0.00	2.38	0.003	2.41
Q61830	Macrophage mannose receptor 1 OS=Mus musculus GN=Mrc1 PE=1 SV=2	39	0.00	2.06	0.003	2.26
P08003	Protein disulfide-isomerase A4 OS=Mus musculus GN=Pdia4 PE=1 SV=3	39	0.00	2.37	0.002	2.62
P09103	Protein disulfide-isomerase OS=Mus musculus GN=P4hb PE=1 SV=2	35	0.00	1.96	0.000	2.26
O88844	Isocitrate dehydrogenase [NADP] cytoplasmic OS=Mus musculus	28	0.00	1.77	0.011	1.80

	GN=Idh1 PE=1 SV=2					
P11835	Integrin beta-2 OS=Mus musculus GN=Itgb2 PE=1 SV=2	28	0.01	2.07	0.005	2.30
P49290	Eosinophil peroxidase OS=Mus musculus GN=Epx PE=1 SV=2	28	0.03	3.78	0.025	4.91
P27046	Alpha-mannosidase 2 OS=Mus musculus GN=Man2a1 PE=1 SV=2	25	0.01	1.52	0.003	1.77
Q8VCT3	Aminopeptidase B OS=Mus musculus GN=Rnpep PE=1 SV=2	24	0.00	1.54	0.009	1.69
P14211	Calreticulin OS=Mus musculus GN=Calr PE=1 SV=1	24	0.00	1.95	0.002	2.08
P05555	Integrin alpha-M OS=Mus musculus GN=Itgam PE=1 SV=2	24	0.01	1.80	0.032	2.22
Q91VI7	Ribonuclease inhibitor OS=Mus musculus GN=Rnh1 PE=1 SV=1	23	0.00	1.55	0.005	1.55
P28867	Protein kinase C delta type OS=Mus musculus GN=Prkcd PE=1 SV=3	22	0.00	1.57	0.002	1.79
Q99KK7	Dipeptidyl peptidase 3 OS=Mus musculus GN=Dpp3 PE=1 SV=2	21	0.00	1.80	0.008	1.83
P97449	Aminopeptidase N OS=Mus musculus GN=Anpep PE=1 SV=4	21	0.00	1.61	0.002	1.70
O09159	Lysosomal alpha-mannosidase OS=Mus musculus GN=Man2b1 PE=1 SV=4	21	0.00	2.16	0.002	2.44
O35744	Chitinase-like protein 3 OS=Mus musculus GN=Chil3 PE=1 SV=2	21	0.01	6.03	0.001	6.96
P39654	Arachidonate 15-lipoxygenase OS=Mus musculus GN=Alox15 PE=1 SV=4	20	0.01	3.52	0.001	3.60
P97494	Glutamate--cysteine ligase catalytic subunit OS=Mus musculus GN=Gclc PE=1 SV=4	20	0.00	2.14	0.001	2.37
Q5SUA5	Unconventional myosin-Ig OS=Mus musculus GN=Myo1g PE=1 SV=1	20	0.00	1.76	0.021	1.76
Q91Z98	Chitinase-like protein 4 OS=Mus musculus GN=Chil4 PE=1 SV=2	20	0.00	4.78	0.000	5.76
P20060	Beta-hexosaminidase subunit beta OS=Mus musculus GN=Hexb PE=1 SV=2	19	0.00	2.43	0.005	2.57
Q8BYW1	Rho GTPase-activating protein 25 OS=Mus musculus GN=Arhgap25 PE=1 SV=2	19	0.00	1.68	0.002	1.73

P40124	Adenylyl cyclase-associated protein 1 OS=Mus musculus GN=Cap1 PE=1 SV=4	19	0.01	1.55	0.010	1.68
Q3TRM8	Hexokinase-3 OS=Mus musculus GN=Hk3 PE=1 SV=2	19	0.00	1.81	0.000	1.95
Q9QXH4	Integrin alpha-X OS=Mus musculus GN=Itgax PE=1 SV=1	18	0.00	1.93	0.004	2.20
Q2VLH6-2	Isoform 2 of Scavenger receptor cysteine-rich type 1 protein M130 OS=Mus musculus GN=Cd163	18	0.00	1.77	0.006	1.71
Q61207	Prosaposin OS=Mus musculus GN=Psap PE=1 SV=2	18	0.02	3.47	0.003	3.45
O89053	Coronin-1A OS=Mus musculus GN=Coro1a PE=1 SV=5	18	0.00	1.80	0.026	1.52
O09131	Glutathione S-transferase omega-1 OS=Mus musculus GN=Gsto1 PE=1 SV=2	17	0.00	1.78	0.005	1.83
P06728	Apolipoprotein A-IV OS=Mus musculus GN=Apoa4 PE=1 SV=3	17	0.02	0.59	0.012	0.63
Q8CIH5	1-phosphatidylinositol 4,5-bisphosphate phosphodiesterase gamma-2 OS=Mus musculus GN=Plcg2 PE=1 SV=1	17	0.00	1.65	0.001	1.62
P70248	Unconventional myosin-If OS=Mus musculus GN=Myo1f PE=1 SV=1	17	0.00	1.59	0.001	1.89
Q5I2A0	Serine protease inhibitor A3G OS=Mus musculus GN=Serpina3g PE=1 SV=2	17	0.00	2.33	0.004	2.47
Q61114	BPI fold-containing family B member 1 OS=Mus musculus GN=Bpifb1 PE=2 SV=4	16	0.00	3.20	0.002	2.82
P18242	Cathepsin D OS=Mus musculus GN=Ctsd PE=1 SV=1	16	0.03	1.82	0.018	1.64
P46978	Dolichyl-diphosphooligosaccharide--protein glycosyltransferase subunit STT3A OS=Mus musculus GN=Stt3a PE=1 SV=1	16	0.00	1.60	0.000	1.90
Q9JII6	Alcohol dehydrogenase [NADP(+)] OS=Mus musculus GN=Akr1a1 PE=1 SV=3	16	0.01	1.54	0.017	1.56
P08074	Carbonyl reductase [NADPH] 2 OS=Mus musculus GN=Cbr2 PE=1 SV=1	16	0.00	0.57	0.004	0.65

P47757	F-actin-capping protein subunit beta OS=Mus musculus GN=Capzb PE=1 SV=3	16	0.00	0.60	0.006	0.34
Q61176	Arginase-1 OS=Mus musculus GN=Arg1 PE=1 SV=1	15	0.03	5.11	0.031	6.17
Q9R111	Guanine deaminase OS=Mus musculus GN=Gda PE=1 SV=1	15	0.00	1.73	0.002	2.19
Q922R8	Protein disulfide-isomerase A6 OS=Mus musculus GN=Pdia6 PE=1 SV=3	15	0.01	1.75	0.000	2.06
Q61035	Histidine--tRNA ligase, cytoplasmic OS=Mus musculus GN=Hars PE=1 SV=2	15	0.00	1.51	0.003	1.67
Q60604	Adseverin OS=Mus musculus GN=Scin PE=1 SV=3	14	0.00	2.16	0.034	1.71
Q11136	Xaa-Pro dipeptidase OS=Mus musculus GN=Pepd PE=1 SV=3	14	0.00	1.80	0.015	1.84
Q9QWR8	Alpha-N-acetylgalactosaminidase OS=Mus musculus GN=Naga PE=1 SV=2	14	0.01	1.72	0.007	1.98
P81117	Nucleobindin-2 OS=Mus musculus GN=Nucb2 PE=1 SV=2	14	0.05	1.99	0.030	2.35
Q9CYN2	Signal peptidase complex subunit 2 OS=Mus musculus GN=Spcs2 PE=1 SV=1	14	0.04	1.54	0.020	1.69
P24369	Peptidyl-prolyl cis-trans isomerase B OS=Mus musculus GN=Ppib PE=1 SV=2	14	0.00	1.60	0.000	1.92
Q9Z110	Delta-1-pyrroline-5-carboxylate synthase OS=Mus musculus GN=Aldh18a1 PE=1 SV=2	14	0.00	1.63	0.005	1.64
Q9Z1G3	V-type proton ATPase subunit C 1 OS=Mus musculus GN=Atp6v1c1 PE=1 SV=4	14	0.00	1.60	0.000	1.66
Q9R1P0	Proteasome subunit alpha type-4 OS=Mus musculus GN=Psma4 PE=1 SV=1	13	0.00	1.54	0.023	1.51
Q61553	Fascin OS=Mus musculus GN=Fscn1 PE=1 SV=4	13	0.00	1.52	0.001	1.61
Q05816	Fatty acid-binding protein, epidermal OS=Mus musculus GN=Fabp5 PE=1 SV=3	13	0.00	2.58	0.003	2.26

Q62000	Mimecan OS=Mus musculus GN=Ogn PE=1 SV=1	13	0.01	0.57	0.002	0.55
Q63918	Serum deprivation-response protein OS=Mus musculus GN=Sdpr PE=1 SV=3	13	0.01	0.57	0.026	0.59
P23492	Purine nucleoside phosphorylase OS=Mus musculus GN=Pnp PE=1 SV=2	13	0.00	1.83	0.009	1.82
Q923B6	Metalloreductase STEAP4 OS=Mus musculus GN=Steap4 PE=1 SV=1	12	0.00	2.05	0.000	2.12
Q91W90	Thioredoxin domain-containing protein 5 OS=Mus musculus GN=Txndc5 PE=1 SV=2	12	0.02	1.55	0.010	1.79
Q99K67	Alpha-aminoadipic semialdehyde synthase, mitochondrial OS=Mus musculus GN=Aass PE=1 SV=1	12	0.00	1.74	0.000	2.03
P01869	Ig gamma-1 chain C region, membrane-bound form OS=Mus musculus GN=Ighg1 PE=1 SV=2	12	0.02	2.07	0.032	1.79
Q9CQI6	Coactosin-like protein OS=Mus musculus GN=Cotl1 PE=1 SV=3	12	0.00	2.48	0.001	2.41
Q9ES52	Phosphatidylinositol 3,4,5-trisphosphate 5-phosphatase 1 OS=Mus musculus GN=Inpp5d PE=1 SV=2	12	0.00	1.63	0.003	1.82
O70570	Polymeric immunoglobulin receptor OS=Mus musculus GN=Pigr PE=1 SV=1	11	0.00	3.02	0.000	3.42
P50404	Pulmonary surfactant-associated protein D OS=Mus musculus GN=Sftpd PE=1 SV=1	11	0.00	2.14	0.014	2.87
Q91WG2	Rab GTPase-binding effector protein 2 OS=Mus musculus GN=Rabep2 PE=1 SV=3	11	0.01	1.61	0.003	1.66
Q61093	Cytochrome b-245 heavy chain OS=Mus musculus GN=Cybb PE=1 SV=1	11	0.00	1.88	0.002	2.30
Q9CXI5	Mesencephalic astrocyte-derived neurotrophic factor OS=Mus musculus GN=Manf PE=1 SV=1	11	0.02	2.22	0.002	2.44

O09046-2	Isoform 2 of L-amino-acid oxidase OS=Mus musculus GN=Il4i1	11	0.00	1.75	0.003	2.05
Q61362	Chitinase-3-like protein 1 OS=Mus musculus GN=Chi3l1 PE=1 SV=3	11	0.00	2.16	0.005	2.45
Q9WTP6	Adenylate kinase 2, mitochondrial OS=Mus musculus GN=Ak2 PE=1 SV=5	11	0.00	1.87	0.013	1.58
Q6ZQI3	Malectin OS=Mus musculus GN=Mlec PE=1 SV=2	11	0.00	1.65	0.002	1.64
P01878	Ig alpha chain C region OS=Mus musculus PE=1 SV=1	10	0.04	3.04	0.007	3.40
P50518	V-type proton ATPase subunit E 1 OS=Mus musculus GN=Atp6v1e1 PE=1 SV=2	10	0.00	1.50	0.002	1.68
Q09014	Neutrophil cytosol factor 1 OS=Mus musculus GN=Ncf1 PE=1 SV=3	10	0.00	1.95	0.001	2.19
O70370	Cathepsin S OS=Mus musculus GN=Ctss PE=1 SV=2	10	0.04	2.99	0.008	3.02
Q922Q8	Leucine-rich repeat-containing protein 59 OS=Mus musculus GN=Lrrc59 PE=1 SV=1	10	0.00	1.55	0.008	1.55
Q9D964	Glycine amidinotransferase, mitochondrial OS=Mus musculus GN=Gatm PE=1 SV=1	10	0.00	2.34	0.004	2.46
Q8BND5	Sulfhydryl oxidase 1 OS=Mus musculus GN=Qsox1 PE=1 SV=1	10	0.01	1.69	0.009	1.82
P01921	H-2 class II histocompatibility antigen, A-D beta chain OS=Mus musculus GN=H2-Ab1 PE=1 SV=1	10	0.01	1.95	0.038	1.79
Q9DBH5	Vesicular integral-membrane protein VIP36 OS=Mus musculus GN=Lman2 PE=1 SV=2	10	0.01	1.72	0.005	1.70
P48025	Tyrosine-protein kinase SYK OS=Mus musculus GN=Syk PE=1 SV=2	10	0.01	1.91	0.003	2.30
P47753	F-actin-capping protein subunit alpha-1 OS=Mus musculus GN=Capza1 PE=1 SV=4	10	0.01	1.57	0.016	1.53
Q01768	Nucleoside diphosphate kinase B OS=Mus musculus GN=Nme2	10	0.00	1.76	0.013	1.79

	PE=1 SV=1					
P45377	Aldose reductase-related protein 2 OS=Mus musculus GN=Akr1b8 PE=1 SV=2	10	0.01	1.70	0.027	1.74
Q9EQU5	Protein SET OS=Mus musculus GN=Set PE=1 SV=1	9	0.00	1.72	0.004	1.66
Q91XA9	Acidic mammalian chitinase OS=Mus musculus GN=Chia PE=1 SV=2	9	0.00	2.65	0.005	2.81
P10605	Cathepsin B OS=Mus musculus GN=Ctsb PE=1 SV=2	9	0.04	2.61	0.010	2.37
P23780	Beta-galactosidase OS=Mus musculus GN=Glb1 PE=1 SV=1	9	0.02	1.69	0.028	1.90
Q6ZWV3	60S ribosomal protein L10 OS=Mus musculus GN=Rpl10 PE=1 SV=3	9	0.01	1.52	0.016	1.63
P16110	Galectin-3 OS=Mus musculus GN=Lgals3 PE=1 SV=3	9	0.02	2.84	0.003	3.39
P68037	Ubiquitin-conjugating enzyme E2 L3 OS=Mus musculus GN=Ube2l3 PE=1 SV=1	9	0.00	1.73	0.036	1.60
Q2TBA3	Mucosa-associated lymphoid tissue lymphoma translocation protein 1 homolog OS=Mus musculus GN=Malt1 PE=1 SV=2	9	0.00	1.70	0.000	1.69
P62918	60S ribosomal protein L8 OS=Mus musculus GN=Rpl8 PE=1 SV=2	9	0.00	1.61	0.021	1.66
P27808	Alpha-1,3-mannosyl-glycoprotein 2-beta-N-acetylglucosaminyltransferase OS=Mus musculus GN=Mgat1 PE=1 SV=1	9	0.00	1.55	0.000	1.79
Q9CYA0	Cysteine-rich with EGF-like domain protein 2 OS=Mus musculus GN=Creld2 PE=1 SV=1	9	0.03	2.18	0.004	2.32
P97821	Dipeptidyl peptidase 1 OS=Mus musculus GN=Ctsc PE=1 SV=1	9	0.00	2.13	0.001	2.43
Q920E5	Farnesyl pyrophosphate synthase OS=Mus musculus GN=Fdps PE=1 SV=1	9	0.00	1.67	0.011	1.62
P34960	Macrophage metalloelastase OS=Mus musculus GN=Mmp12 PE=1 SV=3	9	0.00	3.09	0.001	3.58

Q91YP2	Neurolysin, mitochondrial OS=Mus musculus GN=Nln PE=1 SV=1	9	0.00	1.56	0.003	1.74
P29391	Ferritin light chain 1 OS=Mus musculus GN=Ftl1 PE=1 SV=2	9	0.01	1.60	0.008	1.77
P28352	DNA-(apurinic or apyrimidinic site) lyase OS=Mus musculus GN=Apex1 PE=1 SV=2	9	0.04	1.52	0.009	1.61
Q923T9-3	Isoform 3 of Calcium/calmodulin-dependent protein kinase type II subunit gamma OS=Mus musculus GN=Camk2g	9	0.00	1.52	0.003	1.66
Q80X76	Serine protease inhibitor A3F OS=Mus musculus GN=Serpina3f PE=1 SV=3	9	0.03	3.30	0.008	2.91
P04441-2	Isoform Short of H-2 class II histocompatibility antigen gamma chain OS=Mus musculus GN=Cd74	9	0.01	1.85	0.011	1.82
P62482	Voltage-gated potassium channel subunit beta-2 OS=Mus musculus GN=Kcnab2 PE=1 SV=1	8	0.01	1.77	0.050	1.62
P19253	60S ribosomal protein L13a OS=Mus musculus GN=Rpl13a PE=1 SV=4	8	0.02	1.56	0.048	1.57
P45700	Mannosyl-oligosaccharide 1,2-alpha-mannosidase IA OS=Mus musculus GN=Man1a1 PE=1 SV=1	8	0.00	2.31	0.008	2.28
P06336	Ig epsilon chain C region OS=Mus musculus PE=4 SV=2	8	0.03	2.48	0.034	2.37
P08121	Collagen alpha-1(III) chain OS=Mus musculus GN=Col3a1 PE=1 SV=4	8	0.01	1.65	0.021	1.74
P24638	Lysosomal acid phosphatase OS=Mus musculus GN=Acp2 PE=1 SV=2	8	0.03	1.51	0.007	1.80
P31725	Protein S100-A9 OS=Mus musculus GN=S100a9 PE=1 SV=3	8	0.03	1.52	0.015	1.73
P24452	Macrophage-capping protein OS=Mus musculus GN=Capg PE=1 SV=2	8	0.00	2.65	0.000	2.32
P27870	Proto-oncogene vav OS=Mus musculus GN=Vav1 PE=1 SV=1	8	0.01	1.91	0.042	1.64
Q9ET22	Dipeptidyl peptidase 2 OS=Mus musculus GN=Dpp7 PE=1 SV=2	8	0.00	1.75	0.008	1.86

Q9CXW3	Calcyclin-binding protein OS=Mus musculus GN=Cacybp PE=1 SV=1	8	0.01	1.61	0.018	1.54
Q9WUU7	Cathepsin Z OS=Mus musculus GN=Ctsz PE=1 SV=1	8	0.02	2.88	0.003	3.00
Q9Z0J0	Epididymal secretory protein E1 OS=Mus musculus GN=Npc2 PE=1 SV=1	8	0.00	2.51	0.008	2.49
O70145	Neutrophil cytosol factor 2 OS=Mus musculus GN=Ncf2 PE=1 SV=1	8	0.00	2.26	0.001	2.40
P06797	Cathepsin L1 OS=Mus musculus GN=Ctsl PE=1 SV=2	8	0.03	2.14	0.024	1.99
Q9WVL0	Maleylacetoacetate isomerase OS=Mus musculus GN=Gstz1 PE=1 SV=1	8	0.01	0.66	0.017	0.66
Q61599	Rho GDP-dissociation inhibitor 2 OS=Mus musculus GN=Arhgdib PE=1 SV=3	8	0.00	2.31	0.006	1.85
Q9CQD1	Ras-related protein Rab-5A OS=Mus musculus GN=Rab5a PE=1 SV=1	8	0.00	0.54	0.013	0.66
O88958	Glucosamine-6-phosphate isomerase 1 OS=Mus musculus GN=Gnpda1 PE=1 SV=3	8	0.01	1.64	0.007	1.84
P15532	Nucleoside diphosphate kinase A OS=Mus musculus GN=Nme1 PE=1 SV=1	8	0.00	1.57	0.023	1.59
Q925E7	Serine/threonine-protein phosphatase 2A 55 kDa regulatory subunit B delta isoform OS=Mus musculus GN=Ppp2r2d PE=1 SV=1	8	0.04	1.70	0.045	1.73
Q64524	Histone H2B type 2-E OS=Mus musculus GN=Hist2h2be PE=1 SV=3	8	0.00	0.59	0.009	0.61
P04441	H-2 class II histocompatibility antigen gamma chain OS=Mus musculus GN=Cd74 PE=1 SV=3	8	0.01	2.56	0.034	3.19
P61255	60S ribosomal protein L26 OS=Mus musculus GN=Rpl26 PE=1 SV=1	7	0.02	1.87	0.022	1.83

Q9CXN7	Phenazine biosynthesis-like domain-containing protein 2 OS=Mus musculus GN=Pbld2 PE=1 SV=1	7	0.00	2.26	0.003	2.47
P28798	Granulins OS=Mus musculus GN=Grn PE=1 SV=2	7	0.01	1.64	0.019	1.75
P30412	Peptidyl-prolyl cis-trans isomerase C OS=Mus musculus GN=Ppic PE=1 SV=1	7	0.00	1.53	0.001	1.99
P01837	Ig kappa chain C region OS=Mus musculus PE=1 SV=1	7	0.00	3.01	0.010	2.45
P28651	Carbonic anhydrase-related protein OS=Mus musculus GN=Ca8 PE=1 SV=5	7	0.00	2.01	0.001	2.34
P16045	Galectin-1 OS=Mus musculus GN=Lgals1 PE=1 SV=3	7	0.01	1.74	0.011	1.66
Q9Z0M5	Lysosomal acid lipase/cholesteryl ester hydrolase OS=Mus musculus GN=Lipa PE=1 SV=2	7	0.02	1.64	0.033	1.62
P97501	Dimethylaniline monooxygenase [N-oxide-forming] 3 OS=Mus musculus GN=Fmo3 PE=1 SV=1	7	0.00	0.46	0.002	0.62
Q09200	Beta-1,4 N-acetylgalactosaminyltransferase 1 OS=Mus musculus GN=B4galnt1 PE=1 SV=1	7	0.01	1.53	0.001	1.82
P49935	Pro-cathepsin H OS=Mus musculus GN=Ctsh PE=1 SV=2	7	0.00	1.56	0.003	1.76
P84099	60S ribosomal protein L19 OS=Mus musculus GN=Rpl19 PE=1 SV=1	7	0.01	1.65	0.011	1.79
Q9DCM0	Persulfide dioxygenase ETHE1, mitochondrial OS=Mus musculus GN=Ethe1 PE=1 SV=2	6	0.00	1.79	0.002	1.85
Q60648	Ganglioside GM2 activator OS=Mus musculus GN=Gm2a PE=1 SV=2	6	0.00	2.07	0.001	2.12
Q6R891	Neurabin-2 OS=Mus musculus GN=Ppp1r9b PE=1 SV=1	6	0.00	1.70	0.002	1.75
Q9CPQ1	Cytochrome c oxidase subunit 6C OS=Mus musculus GN=Cox6c PE=1 SV=3	6	0.01	0.62	0.009	0.33
Q566J8	Atypical kinase COQ8B, mitochondrial OS=Mus musculus	6	0.01	1.51	0.018	1.52

	GN=Coq8b PE=1 SV=1					
Q920A5	Retinoid-inducible serine carboxypeptidase OS=Mus musculus GN=Scpep1 PE=1 SV=2	6	0.00	2.03	0.000	2.10
Q9D0B0	Serine/arginine-rich splicing factor 9 OS=Mus musculus GN=Srsf9 PE=1 SV=1	6	0.00	1.56	0.006	1.57
P16675	Lysosomal protective protein OS=Mus musculus GN=Ctsa PE=1 SV=1	6	0.04	2.38	0.016	2.32
P10639	Thioredoxin OS=Mus musculus GN=Txn PE=1 SV=3	6	0.00	1.69	0.029	1.66
Q91VH6	Protein MEMO1 OS=Mus musculus GN=Memo1 PE=1 SV=1	6	0.01	1.56	0.020	1.60
Q60963	Platelet-activating factor acetylhydrolase OS=Mus musculus GN=Pla2g7 PE=2 SV=2	6	0.02	1.69	0.016	1.93
Q6QD59	Vesicle transport protein SEC20 OS=Mus musculus GN=Bnip1 PE=1 SV=1	6	0.00	1.74	0.001	1.57
P57759	Endoplasmic reticulum resident protein 29 OS=Mus musculus GN=Erp29 PE=1 SV=2	6	0.00	1.81	0.002	2.11
Q3UL36	Arginine and glutamate-rich protein 1 OS=Mus musculus GN=Arglu1 PE=1 SV=2	6	0.00	2.05	0.001	1.76
Q8VC28	Aldo-keto reductase family 1 member C13 OS=Mus musculus GN=Akr1c13 PE=1 SV=2	6	0.00	1.54	0.023	1.64
P17897	Lysozyme C-1 OS=Mus musculus GN=Lyz1 PE=1 SV=1	6	0.00	2.21	0.010	2.40
Q05144	Ras-related C3 botulinum toxin substrate 2 OS=Mus musculus GN=Rac2 PE=1 SV=1	6	0.01	1.86	0.043	1.60
Q91XA2	Golgi membrane protein 1 OS=Mus musculus GN=Golm1 PE=1 SV=2	5	0.00	1.81	0.002	2.04
Q61263	Sterol O-acyltransferase 1 OS=Mus musculus GN=Soat1 PE=1 SV=2	5	0.01	1.67	0.002	2.26
P35492	Histidine ammonia-lyase OS=Mus musculus GN=Hal PE=1 SV=1	5	0.00	2.42	0.004	2.33

P50429	Arylsulfatase B OS=Mus musculus GN=Arsb PE=1 SV=3	5	0.00	1.89	0.004	2.00
P55097	Cathepsin K OS=Mus musculus GN=Ctsk PE=1 SV=2	5	0.00	3.41	0.001	3.46
Q9R155	Pendrin OS=Mus musculus GN=Slc26a4 PE=1 SV=1	5	0.00	3.52	0.002	3.72
Q9D1J1	Adaptin ear-binding coat-associated protein 2 OS=Mus musculus GN=Necap2 PE=1 SV=1	5	0.00	1.60	0.000	1.76
Q9JJX6	P2X purinoceptor 4 OS=Mus musculus GN=P2rx4 PE=1 SV=1	5	0.02	2.11	0.003	2.25
P10810	Monocyte differentiation antigen CD14 OS=Mus musculus GN=Cd14 PE=1 SV=1	5	0.02	2.08	0.013	2.47
Q8BU14	Translocation protein SEC62 OS=Mus musculus GN=Sec62 PE=1 SV=1	5	0.02	1.56	0.019	1.57
Q9QZK7	Docking protein 3 OS=Mus musculus GN=Dok3 PE=1 SV=1	5	0.01	1.55	0.005	1.78
Q9D8V7	Signal peptidase complex catalytic subunit SEC11C OS=Mus musculus GN=Sec11c PE=1 SV=3	5	0.00	2.25	0.001	2.52
Q9WVL7	C-X-C motif chemokine 15 OS=Mus musculus GN=Cxcl15 PE=1 SV=1	5	0.00	2.36	0.002	2.49
Q9ESY9	Gamma-interferon-inducible lysosomal thiol reductase OS=Mus musculus GN=Ifi30 PE=1 SV=3	5	0.00	2.72	0.016	3.21
P50543	Protein S100-A11 OS=Mus musculus GN=S100a11 PE=1 SV=1	5	0.00	1.67	0.001	2.21
Q3UMW8	Ceroid-lipofuscinosis neuronal protein 5 homolog OS=Mus musculus GN=Cln5 PE=1 SV=1	5	0.00	1.65	0.007	1.74
P97450	ATP synthase-coupling factor 6, mitochondrial OS=Mus musculus GN=Atp5j PE=1 SV=1	5	0.02	0.63	0.048	0.52
Q9CPT4	Myeloid-derived growth factor OS=Mus musculus GN=Mydgf PE=1 SV=1	5	0.02	1.85	0.018	1.95
Q61017	Guanine nucleotide-binding protein G(I)/G(S)/G(O) subunit gamma-T2 OS=Mus musculus GN=Gngt2 PE=3 SV=2	5	0.01	0.51	0.027	0.64

P08101-2	Isoform IIB2 of Low affinity immunoglobulin gamma Fc region receptor II OS=Mus musculus GN=Fcgr2	5	0.00	2.96	0.004	2.57
P08905	Lysozyme C-2 OS=Mus musculus GN=Lyz2 PE=1 SV=2	5	0.02	2.03	0.004	2.59
Q80SY3	V-type proton ATPase subunit d 2 OS=Mus musculus GN=Atp6v0d2 PE=2 SV=2	4	0.01	1.63	0.018	1.68
Q9QXS6	Drebrin OS=Mus musculus GN=Dbn1 PE=1 SV=4	4	0.01	1.59	0.029	1.53
O08691	Arginase-2, mitochondrial OS=Mus musculus GN=Arg2 PE=1 SV=1	4	0.00	1.89	0.002	2.12
Q64191	N(4)-(beta-N-acetylglucosaminyl)-L-asparaginase OS=Mus musculus GN=Aga PE=1 SV=1	4	0.00	2.02	0.004	2.37
Q9JLM8	Serine/threonine-protein kinase DCLK1 OS=Mus musculus GN=Dclk1 PE=1 SV=1	4	0.00	2.26	0.004	2.28
P97369	Neutrophil cytosol factor 4 OS=Mus musculus GN=Ncf4 PE=1 SV=2	4	0.01	1.87	0.001	2.49
Q8QZV7	Protein asunder homolog OS=Mus musculus GN=Asun PE=1 SV=2	4	0.00	1.76	0.001	1.81
Q8CCH2	NHL repeat-containing protein 3 OS=Mus musculus GN=Nhlrc3 PE=1 SV=1	4	0.02	1.72	0.018	1.77
Q9Z183	Protein-arginine deiminase type-4 OS=Mus musculus GN=Padi4 PE=2 SV=3	4	0.02	1.53	0.008	1.73
Q9CQ80	Vacuolar protein-sorting-associated protein 25 OS=Mus musculus GN=Vps25 PE=1 SV=1	4	0.00	0.55	0.002	0.64
Q9DCJ9	N-acetylneuraminate lyase OS=Mus musculus GN=Npl PE=1 SV=1	4	0.03	1.69	0.023	1.80
Q78IS1	Transmembrane emp24 domain-containing protein 3 OS=Mus musculus GN=Tmed3 PE=1 SV=1	4	0.02	1.92	0.015	2.27
Q8R5J9	PRA1 family protein 3 OS=Mus musculus GN=Arl6ip5 PE=1 SV=2	4	0.00	1.60	0.006	1.67
P20693	Low affinity immunoglobulin epsilon Fc receptor OS=Mus musculus GN=Fcer2 PE=1 SV=1	4	0.03	2.07	0.041	1.83

Q9R0P9	Ubiquitin carboxyl-terminal hydrolase isozyme L1 OS=Mus musculus GN=Uchl1 PE=1 SV=1	4	0.05	0.59	0.036	0.51
P04228	H-2 class II histocompatibility antigen, A-D alpha chain OS=Mus musculus GN=H2-Aa PE=1 SV=1	4	0.02	1.72	0.043	1.65
P51569	Alpha-galactosidase A OS=Mus musculus GN=Gla PE=1 SV=1	4	0.01	1.89	0.006	2.08
Q9D8I1	Marginal zone B- and B1-cell-specific protein OS=Mus musculus GN=Mzb1 PE=1 SV=2	4	0.02	4.41	0.002	4.92
P51437	Cathelicidin antimicrobial peptide OS=Mus musculus GN=Camp PE=1 SV=2	4	0.02	1.53	0.000	1.76
Q9D8S4	Oligoribonuclease, mitochondrial OS=Mus musculus GN=Rexo2 PE=1 SV=2	4	0.00	1.83	0.000	1.77
Q8CIF4	Biotinidase OS=Mus musculus GN=Btd PE=1 SV=2	4	0.02	1.51	0.005	1.77
P34022	Ran-specific GTPase-activating protein OS=Mus musculus GN=Ranbp1 PE=1 SV=2	4	0.02	1.56	0.017	1.51
P07091	Protein S100-A4 OS=Mus musculus GN=S100a4 PE=1 SV=1	4	0.00	1.70	0.004	1.58
Q6NVF4	DNA helicase B OS=Mus musculus GN=Helb PE=1 SV=2	4	0.00	1.56	0.012	1.55
Q9CR09	Ubiquitin-fold modifier-conjugating enzyme 1 OS=Mus musculus GN=Ufc1 PE=1 SV=1	4	0.00	1.71	0.000	1.84
P01807	Ig heavy chain V region X44 OS=Mus musculus PE=1 SV=1	4	0.00	1.72	0.001	1.89
Q571E4	N-acetylgalactosamine-6-sulfatase OS=Mus musculus GN=Galns PE=1 SV=2	4	0.01	1.58	0.028	1.60
Q8R2E9	ERO1-like protein beta OS=Mus musculus GN=Ero1b PE=1 SV=1	4	0.00	1.77	0.002	1.84
P14901	Heme oxygenase 1 OS=Mus musculus GN=Hmox1 PE=1 SV=1	3	0.00	1.59	0.005	1.78
E1U8D0	Protein SOGA1 OS=Mus musculus GN=Soga1 PE=1 SV=3	3	0.01	1.62	0.009	1.61
Q8VBT1	Beta-taxilin OS=Mus musculus GN=Txlnb PE=1 SV=2	3	0.03	0.67	0.001	0.42
Q9EP95	Resistin-like alpha OS=Mus musculus GN=Retnla PE=1 SV=1	3	0.00	4.09	0.007	3.01

O89017	Legumain OS=Mus musculus GN=Lgmn PE=1 SV=1	3	0.00	2.00	0.000	2.24
Q99N69	Leupaxin OS=Mus musculus GN=Lpxn PE=1 SV=2	3	0.01	2.09	0.014	2.00
Q6P8M1	Putative deoxyribonuclease TATDN1 OS=Mus musculus GN=Tatdn1 PE=1 SV=1	3	0.01	1.74	0.003	2.06
Q91XB0	Three-prime repair exonuclease 1 OS=Mus musculus GN=Trex1 PE=1 SV=2	3	0.02	1.54	0.014	1.51
Q9JM51	Prostaglandin E synthase OS=Mus musculus GN=Ptges PE=1 SV=1	3	0.01	1.96	0.006	2.10
Q18PI6	SLAM family member 5 OS=Mus musculus GN=Cd84 PE=1 SV=1	3	0.01	1.81	0.007	1.88
Q9CQ86	Migration and invasion enhancer 1 OS=Mus musculus GN=Mien1 PE=1 SV=1	3	0.00	1.50	0.006	1.51
Q9JI60	Lecithin retinol acyltransferase OS=Mus musculus GN=Lrat PE=1 SV=1	3	0.00	0.65	0.006	0.66
Q9ESP1	Stromal cell-derived factor 2-like protein 1 OS=Mus musculus GN=Sdf2l1 PE=1 SV=2	3	0.03	1.71	0.003	1.95
P01592	Immunoglobulin J chain OS=Mus musculus GN=Jchain PE=1 SV=4	3	0.03	2.45	0.006	2.58
Q9EQX4	Allograft inflammatory factor 1-like OS=Mus musculus GN=Aif1l PE=1 SV=1	3	0.01	1.64	0.041	1.60
Q3U435	Matrix metalloproteinase-25 OS=Mus musculus GN=Mmp25 PE=2 SV=1	3	0.03	1.79	0.049	2.21
Q8VEB4	Group XV phospholipase A2 OS=Mus musculus GN=Pla2g15 PE=1 SV=1	3	0.01	1.59	0.019	1.76
Q06318	Uteroglobin OS=Mus musculus GN=Scgb1a1 PE=1 SV=1	3	0.00	1.92	0.031	1.77
Q9WUL5	Programmed cell death 1 ligand 2 OS=Mus musculus GN=Pdcd1lg2 PE=1 SV=1	3	0.00	1.65	0.008	1.62
P0DN34	NADH dehydrogenase [ubiquinone] 1 beta subcomplex subunit 1 OS=Mus musculus GN=Ndufb1 PE=3 SV=1	3	0.00	0.66	0.015	0.37

P27005	Protein S100-A8 OS=Mus musculus GN=S100a8 PE=1 SV=3	3	0.05	1.54	0.014	1.88
P06330	Ig heavy chain V region AC38 205.12 OS=Mus musculus PE=1 SV=1	3	0.00	2.48	0.004	2.18
Q9DBT5	AMP deaminase 2 OS=Mus musculus GN=Ampd2 PE=1 SV=1	3	0.00	1.80	0.003	2.06
P61953	Guanine nucleotide-binding protein G(I)/G(S)/G(O) subunit gamma-11 OS=Mus musculus GN=Gng11 PE=1 SV=1	3	0.00	0.51	0.017	0.62
A2AAY5	SH3 and PX domain-containing protein 2B OS=Mus musculus GN=Sh3pxd2b PE=1 SV=1	3	0.01	1.74	0.007	2.08
P18524	Ig heavy chain V region RF OS=Mus musculus PE=1 SV=1	3	0.00	2.92	0.002	3.17
P01724	Ig lambda-1 chain V regions MOPC 104E/RPC20/J558/S104 OS=Mus musculus PE=1 SV=1	3	0.01	2.87	0.016	2.78
P07361	Alpha-1-acid glycoprotein 2 OS=Mus musculus GN=Orm2 PE=1 SV=1	3	0.01	1.78	0.041	1.84
P08508	Low affinity immunoglobulin gamma Fc region receptor III OS=Mus musculus GN=Fcgr3 PE=1 SV=1	3	0.01	2.33	0.002	2.72
O08900	Zinc finger protein Aiolos OS=Mus musculus GN=Ikzf3 PE=1 SV=2	2	0.00	1.70	0.009	1.68
Q921V5	Alpha-1,6-mannosyl-glycoprotein 2-beta-N-acetylglucosaminyltransferase OS=Mus musculus GN=Mgat2 PE=1 SV=1	2	0.02	1.55	0.010	1.86
P25085	Interleukin-1 receptor antagonist protein OS=Mus musculus GN=Il1rn PE=2 SV=1	2	0.03	1.60	0.009	2.06
Q91VY9	Zinc finger protein 622 OS=Mus musculus GN=Znf622 PE=1 SV=1	2	0.01	1.70	0.015	1.75
Q8R143	Pituitary tumor-transforming gene 1 protein-interacting protein OS=Mus musculus GN=Pttg1ip PE=1 SV=1	2	0.00	0.63	0.043	0.35
P01633	Ig kappa chain V19-17 OS=Mus musculus GN=Igk-V19-17 PE=1 SV=1	2	0.00	4.53	0.002	3.55

P83877	Thioredoxin-like protein 4A OS=Mus musculus GN=Txnl4a PE=1 SV=1	2	0.01	1.71	0.037	1.59
G5E861	Sodium channel and clathrin linker 1 OS=Mus musculus GN=Sclt1 PE=1 SV=1	2	0.00	2.34	0.014	1.97
O88593	Peptidoglycan recognition protein 1 OS=Mus musculus GN=Pglyrp1 PE=1 SV=1	2	0.00	3.54	0.003	3.60
P08043	Zinc finger protein 2 OS=Mus musculus GN=Zfp2 PE=2 SV=2	2	0.00	1.69	0.020	1.69
Q8C3K6	Sodium/glucose cotransporter 1 OS=Mus musculus GN=Slc5a1 PE=1 SV=1	2	0.01	1.95	0.026	2.22
Q9EQI8	39S ribosomal protein L46, mitochondrial OS=Mus musculus GN=Mrpl46 PE=1 SV=1	2	0.00	1.76	0.002	1.90
P01662	Ig kappa chain V-III region ABPC 22/PC 9245 OS=Mus musculus PE=1 SV=1	2	0.01	1.53	0.001	1.99
P15535	Beta-1,4-galactosyltransferase 1 OS=Mus musculus GN=B4galt1 PE=1 SV=1	2	0.03	2.02	0.015	2.59
P27784	C-C motif chemokine 6 OS=Mus musculus GN=Ccl6 PE=1 SV=1	2	0.04	4.98	0.035	5.87
Q62266	Cornifin-A OS=Mus musculus GN=Sprr1a PE=1 SV=1	2	0.02	4.94	0.013	2.71
Q06185	ATP synthase subunit e, mitochondrial OS=Mus musculus GN=Atp5i PE=1 SV=2	2	0.01	0.53	0.012	0.45
Q9CQA5	Mediator of RNA polymerase II transcription subunit 4 OS=Mus musculus GN=Med4 PE=2 SV=1	2	0.02	0.64	0.008	0.63
Q0P557-3	Isoform 3 of Mitochondria-eating protein OS=Mus musculus GN=Spata18	2	0.00	1.57	0.001	2.03
P17665	Cytochrome c oxidase subunit 7C, mitochondrial OS=Mus musculus GN=Cox7c PE=1 SV=1	2	0.01	0.59	0.042	0.31
O54990	Prominin-1 OS=Mus musculus GN=Prom1 PE=1 SV=1	2	0.00	1.68	0.000	2.21

P29477	Nitric oxide synthase, inducible OS=Mus musculus GN=Nos2 PE=1 SV=1	2	0.00	1.94	0.001	2.67
P01675	Ig kappa chain V-VI region XRPC 44 OS=Mus musculus PE=1 SV=1	2	0.02	1.74	0.042	1.76
Q9Z121	C-C motif chemokine 8 OS=Mus musculus GN=Ccl8 PE=3 SV=1	2	0.04	1.87	0.009	2.34
Q8BGA5	KRR1 small subunit processome component homolog OS=Mus musculus GN=Krr1 PE=2 SV=1	2	0.00	1.68	0.021	1.86
Q3TBT3-2	Isoform 2 of Stimulator of interferon genes protein OS=Mus musculus GN=Tmem173	2	0.00	1.61	0.000	1.80
P31996	Macrosialin OS=Mus musculus GN=Cd68 PE=1 SV=1	2	0.00	2.36	0.003	2.45
Q8R2K1	Fucose mutarotase OS=Mus musculus GN=Fuom PE=1 SV=1	2	0.03	1.65	0.004	1.74
P18528	Ig heavy chain V region 6.96 OS=Mus musculus PE=4 SV=1	2	0.01	1.92	0.010	1.82
P01647	Ig kappa chain V-V region HP 124E1 OS=Mus musculus PE=1 SV=1	2	0.00	2.29	0.002	2.11
P01783	Ig heavy chain V region MOPC 21 (Fragment) OS=Mus musculus PE=1 SV=1	2	0.01	2.37	0.012	2.47
P03980	Ig heavy chain V region TEPC 1017 OS=Mus musculus PE=4 SV=1	2	0.01	2.36	0.013	2.31

Note: A/C refers to the asthma group compared to the control group, while A/S means the asthma group compared to the SCIT group.
