# Supplementary material for: Alteration of lung tissues proteins in birch pollen induced asthma mice before and after SCIT
Source: PLoS One. 2021 Oct 7;16(10):e0258051. doi: 10.1371/journal.pone.0258051 (PMC8496856; doi:10.1371/journal.pone.0258051)
Supplement: S2 Table — (PDF) [file pone.0258051.s005.pdf]

S2 Table. The 277 regression DEPs and previous reports associated with lung diseases

No.	Accession no.	Description	Previous reports
1	P27784	C-C motif chemokine 6 OS=Mus musculus GN=Ccl6 PE=1 SV=1	1, 2, 4, 6
2	P31996	Macrosialin OS=Mus musculus GN=Cd68 PE=1 SV=1	1
3	Q18PI6	SLAM family member 5 OS=Mus musculus GN=Cd84 PE=1 SV=1	1
4	O35744	Chitinase-like protein 3 OS=Mus musculus GN=Chil3 PE=1 SV=2	1, 4

5	Q91Z98	Chitinase-like protein 4 OS=Mus musculus GN=Chil4 PE=1 SV=2	1, 3, 6
6	P18242	Cathepsin D OS=Mus musculus GN=Ctsd PE=1 SV=1	1, 7
7	P55079	Cathepsin K OS=Mus musculus GN=Ctsk PE=1 SV=2	1, 6
8	Q9QXH4	Integrin alpha-X OS=Mus musculus GN=Itgax PE=1 SV=1	1
9	P34960	Macrophage metalloelastase OS=Mus musculus GN=Mmp12 PE=1 SV=3	1, 3, 4
10	Q9EP95	Resistin-like alpha OS=Mus musculus GN=Retnla PE=1 SV=1	1, 4
11	P08071	Lactotransferrin OS=Mus musculus GN=Ltf PE=1 SV=4	1
12	Q06318	Uteroglobin OS=Mus musculus GN=Scgb1a1 PE=1 SV=1	1, 3
13	Q9Z0J0	Epididymal secretory protein E1 OS=Mus musculus GN=Npc2 PE=1 SV=1	2
14	Q61176	Arginase-1 OS=Mus musculus GN=Arg1 PE=1 SV=1	2, 3, 4, 5
15	Q61830	Macrophage mannose receptor 1 OS=Mus musculus GN=Mrc1 PE=1 SV=2	2, 5
16	P31725	Protein S100-A9 OS=Mus musculus GN=S100a9 PE=1 SV=3	2, 8
17	O89017	Legumain OS=Mus musculus GN=Lgmn PE=1 SV=1	2
18	P08905	Lysozyme C-2 OS=Mus musculus GN=Lyz2 PE=1 SV=2	2
19	Q9D7Z6	Calcium-activated chloride channel regulator 1 OS=Mus musculus GN=Clca1 PE=1	3
20	O70570	Polymeric immunoglobulin receptor OS=Mus musculus GN=Pigr PE=1 SV=1	3
21	O88593	Peptidoglycan recognition protein 1 OS=Mus musculus GN=Pglyrp1 PE=1 SV=1	3
22	P49290	Eosinophil peroxidase OS=Mus musculus GN=Epx PE=1 SV=2	3
23	Q9Z121	C-C motif chemokine 8 OS=Mus musculus GN=Ccl8 PE=3 SV=1	3, 7
24	P31725	Protein S100-A9 OS=Mus musculus GN=S100a9 PE=1 SV=3	3, 4, 5, 6, 8

25	O08691	Arginase-2, mitochondrial OS=Mus musculus GN=Arg2 PE=1 SV=1	4, 5
26	Q9R155	Pendrin OS=Mus musculus GN=Slc26a4 PE=1 SV=1	4
27	Q60604	Adseverin OS=Mus musculus GN=Scin PE=1 SV=3	4
28	Q5I2A0	Serine protease inhibitor A3G OS=Mus musculus GN=Serpina3g PE=1 SV=2	4, 5
29	O70145	Neutrophil cytosol factor 2 OS=Mus musculus GN=Ncf2 PE=1 SV=1	4
30	P97369	Neutrophil cytosol factor 4 OS=Mus musculus GN=Ncf4 PE=1 SV=2	5
31	P25085	Interleukin-1 receptor antagonist protein OS=Mus musculus GN=Il1rn PE=2 SV=1	5, 8
32	Q3TRM8	Hexokinase-3 OS=Mus musculus GN=Hk3 PE=1 SV=2	5
33	P08121	Collagen alpha-1(III) chain OS=Mus musculus GN=Col3a1 PE=1 SV=4	5
34	O89017	Legumain OS=Mus musculus GN=Lgmn PE=1 SV=1	6
35	Q9WUU7	Cathepsin Z OS=Mus musculus GN=Ctsz PE=1 SV=1	6
36	Q05144	Ras-related C3 botulinum toxin substrate 2 OS=Mus musculus GN=Rac2 PE=1 SV=1	6
37	P27005	Protein S100-A8 OS=Mus musculus GN=S100a8 PE=1 SV=3	8
38	Q61362	Chitinase-3-like protein 1 OS=Mus musculus GN=Chi3l1 PE=1 SV=3	8
39	P10605	Cathepsin B OS=Mus musculus GN=Ctsb PE=1 SV=2	8

Reference:

1. Shuto T, Kamei S, Nohara H, et al. Pharmacological and genetic reappraisals of protease and oxidative stress pathways in a mouse model of obstructive lung diseases. *Sci Rep.*

2016;6:39305.
2. Kamata T, Jin H, Giblett S, et al. The cholesterol-binding protein NPC2 restrains recruitment of stromal macrophage-lineage cells to early-stage lung tumours. *EMBO Mol Med.* 2015;7(9):1119-1137.
3. Louten J, Mattson JD, Malinao MC, et al. Biomarkers of disease and treatment in murine and cynomolgus models of chronic asthma. *Biomark Insights.* 2012;7:87-104.
4. Di Valentin E, Crahay C, Garbacki N, et al. New asthma biomarkers: lessons from murine models of acute and chronic asthma. *Am J Physiol Lung Cell Mol Physiol.* 2009;296(2):L185-197.
5. Gonzalez-Juarrero M, Kingry LC, Ordway DJ, et al. Immune response to Mycobacterium tuberculosis and identification of molecular markers of disease. *Am J Respir Cell Mol Biol.* 2009;40(4):398-409.
6. Camateros P, Kanagaratham C, Henri J, Sladek R, Hudson TJ, Radzioch D. Modulation of the allergic asthma transcriptome following resiquimod treatment. *Physiol Genomics.* 2009;38(3):303-318.
7. Kan M, Shumyatcher M, Himes BE. Using omics approaches to understand pulmonary diseases. *Respir Res.* 2017;18(1):149.
8. Cao C, Li W, Hua W, et al. Proteomic analysis of sputum reveals novel biomarkers for various presentations of asthma. *J Transl Med.* 2017;15(1):171.
