# Supplementary material for: Alteration of lung tissues proteins in birch pollen induced asthma mice before and after SCIT
Source: PLoS One. 2021 Oct 7;16(10):e0258051. doi: 10.1371/journal.pone.0258051 (PMC8496856; doi:10.1371/journal.pone.0258051)
Supplement: S3 Table — (PDF) [file pone.0258051.s006.pdf]

S3 Table. The details of the DEPs in the leukocyte extravasation signaling

Symbol	Gene Name	Accession	Location	Type(s)
ITGAM	integrin subunit alpha M	P05555	Plasma Membrane	transmembrane receptor
ITGB2	integrin subunit beta 2	P11835	Plasma Membrane	transmembrane receptor
MMP12	matrix metallopeptidase 12	P34960	Extracellular Space	peptidase
NCF1	neutrophil cytosolic factor 1	Q09014	Cytoplasm	enzyme
NCF2	neutrophil cytosolic factor 2	O70145	Cytoplasm	enzyme
NCF4	neutrophil cytosolic factor 4	P97369	Cytoplasm	enzyme
RAC2	Rac family small GTPase 2	Q05144	Cytoplasm	enzyme
VAV1	vav guanine nucleotide exchange factor 1	P27870	Nucleus	transcription regulator
