# Supplementary material for: Alteration of lung tissues proteins in birch pollen induced asthma mice before and after SCIT
Source: PLoS One. 2021 Oct 7;16(10):e0258051. doi: 10.1371/journal.pone.0258051 (PMC8496856; doi:10.1371/journal.pone.0258051)
Supplement: S1 File — (DOCX) [file pone.0258051.s007.docx]

# Supporting information and materials and methods

## Animal models

Female BALB/c mice (5-6 weeks) were purchased from Beijing Vital River Laboratory Animal Technology Co in Beijing, China. The animals were housed (3-4 mice per cage) in specific pathogen-free (SPF) conditions with a 12 h light/dark cycle and constant room temperature.

Standard birch pollen extract (BPE) was kindly provided by the Allergy Department of Peking Union Medical College Hospital in Beijing, China. All protein concentrations were determined using a Pierce BCA protein assay kit (Thermo Fisher Scientific, Waltham, Mass).

Previously, our group have reported on this mouse model and its SCIT protocols in detail [15]. This model suggested Chinese birch pollen with an alum adjuvant successfully induced airway inflammation in mice and that BPE related SCIT provided long-term therapeutic benefits.

To further explore the asthma versus the control profiles, Female BALB/c mice (N=10-12, per group) were primary sensitized with 100 μL (25 μg/μL) birch pollen extract (BPE) or phosphate buffered saline (PBS) mixed with 100 μL alum (Imject Alum, Thermo, USA) by subcutaneous injection on days 0, 7 and 14. The mice received nebulized PBS or 0.1% BPE in PBS 3 times for 30 min on days 21-23. On day 24, the mice were anesthetized by intraperitoneal injection of 0.3-0.6 ml 1% pentobarbital until the pain reflex disappeared, then sacrificed for sample preparation (Fig 1B). We gave the mice euthanasia by anesthesia overdose. The evidence of euthanasia based on moribund appearance. The pain for the mice and method to calm is mild, transient or without pain.

To further explore the asthma versus SCIT profiles, the SCIT group mice (N=10-12, per group) were sensitized and challenged in the same manner as the asthma group and then subcutaneously injected with 150 μL (2 μg/μL)) BPE adsorbed to 50 μL alum on days 30, 37, 44, 51, 58, 65, 72 and 79. On days 86-88, the mice received nebulized 0.1% BPE in PBS for 30 min per day before they were killed, and the samples were collected (Fig 1B). During the whole process, there was no accidental death of mice based on our careful experimental operations.

According to the results of similar experiments, the estimated standard deviation is about 0.115, and the difference between the two groups is about 0.25. The calculation formula of sample size in animal experiment: N=2*[(1.96+1.282)×S/X]^2^. The sample size of mice is usually 10 mice, considering the death factor in the experiment. Thus, the exact number of experimental units allocated to each group is 10 mice, and the total number in our experiment is 50 mice.

## Inflammatory cells in bronchoalveolar lavage fluids (BALF)

Inflammatory cells in bronchoalveolar lavage fluids (BALF) were collected by a tracheal cannula. The bronchial tube was lavaged three times with 0.8 mL PBS to collect lavage fluid. The obtained BALF was centrifuged at 4000 × *g* for 10 min at 4 °C. The supernatant was discarded. The pellets of the BALF were resuspended with 1 mL PBS. Then, a Siemens automatic blood analyzer (ADVIA2120) was used to analyze inflammatory cells.

## Lung tissue sample preparation

After the collection of BALF, the pulmonic vasculature was flushed with PBS until the lung tissues became white and the remaining blood was cleared. Then, clean and fresh lung tissues were equally divided into 2 parts, one piece was used for histological examination and immunohistochemistry while the other half was used for proteomics analysis. In short, one-sixth of the whole lung tissue was taken for proteomics analysis from individual mice at each time point.

## Histological examination

The lungs were fixed with 10% formaldehyde and embedded in paraffin and then cut into slices for staining with hematoxylin & eosin (H&E). H&E staining was used to evaluate the infiltration of inflammatory cells around the airways and blood vessels.

## Immunohistochemistry (IHC)

The following antibodies were purchased: anti-CD11b (ab133357), anti-CD18 (ab185723), and anti-RAC2 (ab2244) (Abcam, Cambridge, UK). MMP12 (PA5-13181) and Vav1 (Tyr174) (PA5-36699) were purchased from Thermo Fisher Scientific (Thermo Fisher Scientific, Waltham, Mass). The goat anti-rabbit IgG (HRP) (GB23303) and rabbit anti-goat IgG (HRP) (GB23204) secondary antibodies were purchased from Servicebio (Servicebio, Wuhan, China). IHC was performed on formalin-fixed and paraffin-embedded lung tissue strictly according to the protocols. The whole lung tissue was cut into microslides, deparaffinized, rehydrated and then treated with EDTA antigen retrieval solution (pH 8.0) in a microwave oven. After 3 PBS washes, the slides were treated with 3% hydrogen peroxide solution for 25 min at room temperature. For most slides, blocking buffer (3% BSA-PBST) was added and incubated for 30 min at room temperature, while the slides that were to be stained with anti-RAC2 antibodies required rabbit serum as the blocking buffer. Then, the slides were incubated with anti-CD11b (ITGAM) (1:1000), anti-CD18 (ITGB2) (1:200), anti-RAC2 (1:1500), MMP12 (1:150), or phospho-VAV1 (1:200) overnight at 4 °C. The secondary antibodies were added to the slides for 50 min at 37 °C, and then the slides were stained according to the protocols. The slides were scanned and digitized by using a 3D HISTECH Panoramic tissue slice scanner (https://www.3dhistech.com). We utilized the 3D HISTECH Quant center 2.1 software to evaluate the H-score of the stained sections. The H-score, also known as the histochemistry score, is a semi-quantitative histological scoring method that transfers the number of positive cells and the staining intensity of each section into corresponding values. H-score = ∑(Pi×I) = (percentage of cells of weak intensity ×1)+(percentage of cells of moderate intensity ×2)+percentage of cells of strong intensity ×3), where I = intensity of staining and Pi = percentage of stained cells. (H-score range: 0-300; negative: blue; weak intensity: light yellow; moderate intensity: pale brown; and strong intensity: dark brown).

## Protein digestion and TMT labeling

The excised lung tissues from individual mice were sonicated and lysed with buffer (7 M urea; 2 M thiourea; 5 mM DTT; 50 mM Tris), containing DNase, RNase and protease inhibitor. The lysates were centrifuged at 14,000 *g* for 15 min at 4 °C, and the supernatant protein concentration was determined using a Pierce BCA protein assay kit (Thermo Fisher Scientific, Waltham, Mass). Each group of samples was mixed into three sample pools for further study. Each sample pool (200 μL) was reduced with 20 mM dithiothreitol (DTT) for 5 min at 95 °C and then alkylated with 55 mM IAM for 45 min at room temperature in the dark. The samples were purified with ice-cold acetone for 30 min at -20 °C. The samples were centrifuged at 14,000 × g for 10 min and then air-dried. Tris solution (20 mM) was added to each sample to resolve the pellets. The filter aided sample preparation (FASP) method was applied and the proteins were digested with a mixture of sample pool lysate and trypsin at a 1:50 (w/w) ratio overnight at 37°C. To purify the peptide for TMT labeling, the digested products were undergoing solid phase extraction using a Waters Oasis C18 (Solid Phase Extraction Column) and then dried down and stored at -80 °C.

The experimental procedure for Thermo Scientific™ Tandem Mass Tag™ (TMT™) reagent labeling was performed strictly according to the manufacturer’s instructions. In brief, 100ug peptides power was reconstituted in 100 mM triethylammonium bicarbonate (TEAB). TMT 10-plex labeling reagents were randomly distributed to label the different sample pools. Each group of lung samples (the control group, the asthma group and the SCIT group; N=9) was mixed separately into three sample pools. TMT-127N, TMT-128C, and TMT-129N were used for the PBS control group sample pools; TMT-129C, TMT-130N, and TMT-130C were used for the asthma group sample pools; and TMT-126, TMT-128N, and TMT-127C were used for the SCIT group sample pools. A mixed sample pool for raw data normalization was prepared from each individual sample pool and labeled with TMT-131 (Fig 1C).

## High performance liquid chromatography (HPLC)

The TMT-labeled peptide samples were mixed with equal volume and fractioned with the high-RP HPLC column from Waters (4.6 mm × 250 mm, C18, 3 μm) with a flow rate of 0.7 mL/min. UV absorbance was detected at 215 nm. The procedure was 60 min. The gradient elution buffer consisted of ddH2O (pH 10.0) and 98% acetonitrile (pH 10.0). The fractioned elutes were collected into 60 microtubes. The fractions were combined into 20 samples as follows: 1,21,41; 2,22,42; and so on.

## LC-MS/MS

The fractionated peptides were resolved in 0.1% FA, and then delivered to LC-MS/MS for identification. A Thermo Scientific™ Orbitrap™ Fusion™ Lumos mass spectrometer (Thermo Fisher Scientiﬁc, Waltham, MA, USA) equipped NanoEasy 1000 liquid chromatography system (Thermo Scientiﬁc, Waltham, MA, USA) were adopted. The column was Acclaim PepMap100, C18, 3 μm, 100A, 75 μm IDx 25 cm (Thermo Fisher Scientiﬁc, Waltham, MA, USA). The online elution gradient was set as follows: Phase A: H_2_O, 0.1% FA; Phase B: ACN, 0.1% FA. Linear gradients of 0-2% B for 2 min; 2-6% B for 2 min; 6-10% B for 12 min; 10-20% B for 36 min; 20-28% B for 7 min; 28-95% B for 1 min and finally 95% B for 2 min with a flow rate of 600 nL/min. The ion source parameters were set as follows: spray voltage: 2.2 kV; capillary temperature: 320℃. In the full mass mode: resolution: 60000; full scan AGC target: 1e6; full scan max. IT: 20 msec; scan range: 300-1500 m/z. In the dd-MS2 mode: Resolution: 50000; AGC target: 1e4; Max. IT: 86 msec; Intensity threshold: 5e4; Fragmentation methods: HCD.

## TMT data analysis

The raw data of the LC-MS/MS were analyzed by using Proteome Discoverer 2.1 (Thermo Scientific, Waltham, USA). It was set up to search the SwissProt-Mouse proteome database (Released on April 18^th^, 2017). The search criteria were set as follow parameters: peptide tolerance of 20 ppm; product ion tolerance of 0.05 Da; carbamidomethylation of cysteine and TMT 10-plex were used as fixed modification; and oxidation of methionine was set as the dynamic modification. Protein identifications were accepted if they could be established at greater than 95.0% probability to achieve a false discovery rate (FDR) less than 1.0% and filtered with no less two unique peptides.

## Bioinformatics analysis

SIMAC (version 15) was used to perform the principal component analysis (PCA). The differentially expressed proteins (DEPs) were screened with fold change≥1.5 or ≤0.67 and *P* values ≤0.05. The Venn image of the DEPs was prepared by the platform ([*http://bioinformatics.psb.ugent.be/webtools/Venn/*](http://bioinformatics.psb.ugent.be/webtools/Venn/)). Gene ontology (GO) classification analysis was performed to analyze the biological process (BP), molecular function (MF) and cellular component (CC) using the PANTHER platform ([*http://www.pantherdb.org/*](http://www.pantherdb.org/)). For the ingenuity pathway analysis, we used IPA software (Released on 2018-10-9) to identify the top canonical pathways and the diseases and biofunctions. Statistical significance of the canonical pathways and the diseases and biofunctions were based on Z-scores. Z-scores ≥ 2.0 or ≤ -2.0 suggested that the pathway was positively activated or negatively activated, respectively.
