# Supplementary material for: Alteration of lung tissues proteins in birch pollen induced asthma mice before and after SCIT
Source: PLoS One. 2021 Oct 7;16(10):e0258051. doi: 10.1371/journal.pone.0258051 (PMC8496856; doi:10.1371/journal.pone.0258051)
Supplement: S1 Checklist — (PDF) [file pone.0258051.s008.pdf]

NOTE: Please save this file locally before filling in the table, DO NOT work on the file within your internet browser as changes will not be saved. Adobe Acrobat Reader (available free here) is recommended for completion.

ARRIVE

The ARRIVE guidelines 2.0: author checklist

The ARRIVE Essential 10

These items are the basic minimum to include in a manuscript. Without this information, readers and reviewers cannot assess the reliability of the findings.

Item		Recommendation	Section/line number, or reason for not reporting
**Study design**	1	For each experiment, provide brief details of study design including:	
		a. The groups being compared, including control groups. If no control group has been used, the rationale should be stated.	in methods section, line 130-143 and in supporting information and materials and methods, line 633-655.
		b. The experimental unit (e.g. a single animal, litter, or cage of animals).	in supporting information and materials and methods, line 650 and line 634.
**Sample size**	2	a. Specify the exact number of experimental units allocated to each group, and the total number in each experiment. Also indicate the total number of animals used.	in supporting information and materials and methods, line 656-660.
		b. Explain how the sample size was decided. Provide details of any *a priori* sample size calculation, if done.	in supporting information and materials and methods, line 656-660.
**Inclusion and exclusion criteria**	3	a. Describe any criteria used for including and excluding animals (or experimental units) during the experiment, and data points during the analysis. Specify if these criteria were established *a priori*. If no criteria were set, state this explicitly.	Mice were grouped according to a random number.
		b. For each experimental group, report any animals, experimental units or data points not included in the analysis and explain why. If there were no exclusions, state so.	During the whole process, there was no accidental death of mice based on our careful experimental operations.
		c. For each analysis, report the exact value of *n* in each experimental group.	We have included all the experimental data. We have reported the exact value of sample number in each experimental group.
**Randomisation**	4	a. State whether randomisation was used to allocate experimental units to control and treatment groups. If done, provide the method used to generate the randomisation sequence.	Mice were grouped according to a random number table.
		b. Describe the strategy used to minimise potential confounders such as the order of treatments and measurements, or animal/cage location. If confounders were not controlled, state this explicitly.	To minimise potential confounders, mice were treated equally both in control and treatment groups.
**Blinding**	5	Describe who was aware of the group allocation at the different stages of the experiment (during the allocation, the conduct of the experiment, the outcome assessment, and the data analysis).	The treatment and subsequent analyses were performed in a blind fashion for all groups.
**Outcome measures**	6	a. Clearly define all outcome measures assessed (e.g. cell death, molecular markers, or behavioural changes).	in supporting information and materials and methods, line 614-625.
		b. For hypothesis-testing studies, specify the primary outcome measure, i.e. the outcome measure that was used to determine the sample size.	not mentioned in the manuscript.
**Statistical methods**	7	a. Provide details of the statistical methods used for each analysis, including software used.	in methods section, line 202-205.
		b. Describe any methods used to assess whether the data met the assumptions of the statistical approach, and what was done if the assumptions were not met.	in methods section, line 202-205.
**Experimental animals**	8	a. Provide species-appropriate details of the animals used, including species, strain and substrain, sex, age or developmental stage, and, if relevant, weight.	in supporting information and materials and methods, line 614-625.
		b. Provide further relevant information on the provenance of animals, health/immune status, genetic modification status, genotype, and any previous procedures.	in supporting information and materials and methods, line 614-625.
**Experimental procedures**	9	For each experimental group, including controls, describe the procedures in enough detail to allow others to replicate them, including:	in supporting information and materials and methods
		a. What was done, how it was done and what was used.	in supporting information and materials and methods, line 622-781.
		b. When and how often.	in supporting information and materials and methods, line 622-781.
		c. Where (including detail of any acclimatisation periods).	
		d. Why (provide rationale for procedures).	in supporting information and materials and methods, line 622-781.
**Results**	10	For each experiment conducted, including independent replications, report:	
		a. Summary/descriptive statistics for each experimental group, with a measure of variability where applicable (e.g. mean and SD, or median and range).	in results rection.
		b. If applicable, the effect size with a confidence interval.	Not Applied.

The Recommended Set

These items complement the Essential 10 and add important context to the study. Reporting the items in both sets represents best practice.

Item		Recommendation	Section/line number, or reason for not reporting
**Abstract**	11	Provide an accurate summary of the research objectives, animal species, strain and sex, key methods, principal findings, and study conclusions.	in abstract section, line 31-61.
**Background**	12	a. Include sufficient scientific background to understand the rationale and context for the study, and explain the experimental approach.	in introduction section
		b. Explain how the animal species and model used address the scientific objectives and, where appropriate, the relevance to human biology.	in introduction section
**Objectives**	13	Clearly describe the research question, research objectives and, where appropriate, specific hypotheses being tested.	in abstract section, line 32-35.
**Ethical statement**	14	Provide the name of the ethical review committee or equivalent that has approved the use of animals in this study, and any relevant licence or protocol numbers (if applicable). If ethical approval was not sought or granted, provide a justification.	in methods section, line 116-123.
**Housing and husbandry**	15	Provide details of housing and husbandry conditions, including any environmental enrichment.	in supporting information and materials and methods, line 615-618
**Animal care and monitoring**	16	a. Describe any interventions or steps taken in the experimental protocols to reduce pain, suffering and distress.	in supporting information and materials and methods, line 615-618
		b. Report any expected or unexpected adverse events.	in supporting information and materials and methods, line 655-656.
		c. Describe the humane endpoints established for the study, the signs that were monitored and the frequency of monitoring. If the study did not have humane endpoints, state this.	in supporting information and materials and methods, line 647-649.
**Interpretation/ scientific implications**	17	a. Interpret the results, taking into account the study objectives and hypotheses, current theory and other relevant studies in the literature.	in disscusion and introduction section
		b. Comment on the study limitations including potential sources of bias, limitations of the animal model, and imprecision associated with the results.	in disscusion section
**Generalisability/ translation**	18	Comment on whether, and how, the findings of this study are likely to generalise to other species or experimental conditions, including any relevance to human biology (where appropriate).	in disscusion and introduction section.
**Protocol registration**	19	Provide a statement indicating whether a protocol (including the research question, key design features, and analysis plan) was prepared before the study, and if and where this protocol was registered.	in author contribution section, line 827-831.
**Data access**	20	Provide a statement describing if and where study data are available.	in availability of data and materials section, line 818-825.
**Declaration of interests**	21	a. Declare any potential conflicts of interest, including financial and non-financial. If none exist, this should be stated.	in declaration of conflict of interest section,line 800-801
		b. List all funding sources (including grant identifier) and the role of the funder(s) in the design, analysis and reporting of the study.	in funding Sources section, line 804-805.
